# Goos–Hänchen effect singularities in transdimensional plasmonic films

**DOI:** 10.1515/nanoph-2025-0266

**Published:** 2025-09-18

**Authors:** Svend-Age Biehs, Igor V. Bondarev

**Affiliations:** Institut für Physik, Carl von Ossietzky Universität, 26111 Oldenburg, Germany; Department of Mathematics & Physics, 3066 North Carolina Central University, Durham, NC 27707, USA

**Keywords:** Goos–Hänchen effect, transdimensional plasmonic films, topologically protected singularities

## Abstract

We identify and classify topologically protected singularities for the reflection coefficient of transdimensional plasmonic systems. Originating from nonlocal electromagnetic response due to vertical electron confinement in the system, such singularities lead to lateral (angular) Goos–Hänchen shifts on the millimeter (milliradian) scale in the visible range, greatly exceeding those reported previously for artificially designed metasurfaces, offering new opportunities for quantum material development.

## 1 Introduction

It has been known that the reflection of a linearly polarized optical beam of finite transverse extent incident on a plane surface does not exactly follow the Snell’s law of geometrical optics [1], [2]. Instead, the reflected beam experiences slight lateral in-plane displacement and angular deflection in the plane of incidence – the phenomenon commonly referred to as the Goos–Hänchen (GH) effect. Originating from the spatial dispersion of reflection or transmission coefficients due to the finite transverse size of the beam (and so nonlocal in its nature), the GH effect occurs for both reflected and refracted light in realistic optical systems. It was observed in a variety of systems (see Ref. [2]) including plasmonic metamaterials [3], graphene [4], and even neutron scattering experiments [5]. These days the effect attracts much attention as well [6], [7], [8] due to the new generation of materials being available – quantum nanomaterials of reduced dimensionality, materials that can enhance nonlocal subwavelength light propagation to offer new directions for quantum optics, quantum nanophotonics, and quantum computing application development [9], [10].

Plasmonic transdimensional (TD) quantum materials are atomically thin metal (or semiconductor) films of precisely controlled thickness [11]. Currently available due to great progress in nanofabrication techniques [12], [13], [14], [15], [16], [17], [18], such materials offer high tailorability of their electronic and optical properties not only by altering their chemical and/or electronic composition (stoichiometry, doping) but also by merely varying their thickness (number of monolayers) [19], [20], [21], [22], [23], [24], [25], [26], [27], [28], [29], [30], [31], [32]. They provide a new regime – transdimensional, in between three (3D) and two (2D) dimensions, turning into 2D as the film thickness tends to zero. In this regime, the strong vertical quantum confinement makes the linear electromagnetic (EM) response of the TD film nonlocal, or spatially dispersive, and the degree of nonlocality can be controlled by varying the film thickness [22], [26]. That is what makes plasmonic TD films indispensable for studies of the nonlocal light–matter interactions at the nanoscale [28], [29], [30], [31], [32].

The properties of the TD plasmonic films can be explained by the confinement-induced nonlocal EM response theory [22], [23] built on the Keldysh–Rytova (KR) electron interaction potential [33]. The theory is verified experimentally in a variety of settings [13], [14], [15], [16]. It accounts for vertical electron confinement due to the presence of substrate and superstrate of dielectric permittivities less than that of the film, whereby the thickness of the film becomes a parameter to control its nonlocal EM response. The KR model covers both ultrathin films of thickness much less than the half-wavelength of incoming light radiation and conventional films as thick as a few optical wavelengths [22], [23]. The nonlocal EM response of TD plasmonic systems enables a variety of new effects such as thickness-controlled plasma frequency red shift [15], low-temperature plasma frequency dropoff [16], plasma mode degeneracy lifting [26], a series of quantum-optical [28], [34] and nonlocal magneto-optical effects [23], as well as thermal and vacuum field fluctuation effects responsible for near-field heat transfer [14], [30] and Casimir interaction phenomena [31], [32].

Here, we focus on the confinement-induced nonlocality of the EM response of the TD plasmonic film to study theoretically the GH effect for an incident laser beam. Using the nonlocal KR model, analytical calculations and numerical analysis, we identify and classify topologically protected singularities for the nonlocal reflection coefficient of the system. Such singularities are shown to lead to giant lateral and angular GH shifts in the millimeter and milliradian range, respectively, to greatly exceed those of microscale reported for beams of finite transverse extent with no material-induced nonlocality [2], [3], [4], [5], [6], [7]. They appear in TD materials with broken in-plane reflection symmetry (substrate and superstrate of different dielectric permittivities) where due to the confinement-induced nonlocality the eigenmode degeneracy is lifted to create the points of topological darkness in the visible range not existing in standard local Drude materials.

## 2 The Goos–Hänchen shift

**Figure 1:**
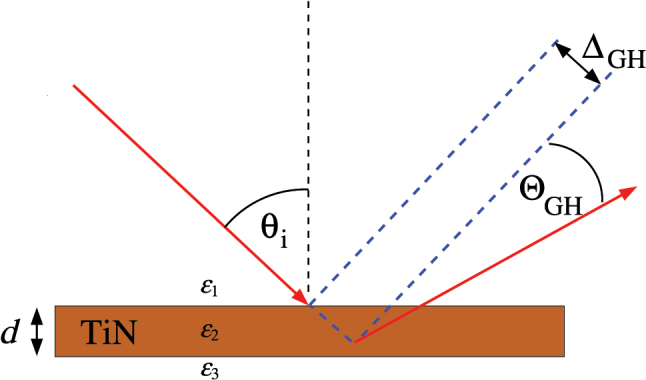
GH shifts Δ_GH_ and Θ_GH_ with TiN plasmonic slab. A detector placed a distance *l* from the slab surface measures the shift Δ_total_ = Δ_GH_ + *l* tan(Θ_GH_) ≈ Δ_GH_ + *l* Θ_GH_.

The theory of the GH shift was originally formulated by Artmann back in 1948 [35]. The geometry of the effect is shown in Figure 1. After reflectance at an interface, the lateral and angular shifts of an incoming *p*-polarized wave in medium 1 (refractive index *n*_1_) are given by [2], [35], [36], [37]

(1) $$\Delta_{\text{GH}}=n_{1}\cos\theta_{i}\frac{\partial \varphi_{\;p}}{\partial k}$$

and

(2) $$\Theta_{\text{GH}}=-\frac{\theta_{0}^{2}}{2}k_{0}n_{1}\frac{\cos\theta_{i}}{\left|R_{p}\right|}\frac{\partial |R_{p}|}{\partial k},$$

respectively. Here, *k* = *k*_0_ *n*_1_sin*θ*_*i*_ is the wavevector in-plane projection, *θ*_*i*_ is the angle of incidence, *k*_0_ = *ω*/*c*, and *θ*_0_ = 2/(*w*_0_ *k*_0_ *n*_1_) is the angular spread of an incident Gaussian light beam of waist *w*_0_. The *p*-wave reflection coefficient is written as $R_{p}=|R_{p}|{\text{e}}^{\text{i}\varphi_{p}}$ in the complex exponential form. Both shifts can be seen being spatially dispersive, with Δ_GH_ being sensitive to reflectivity phase jumps and Θ_GH_ to zero reflection itself so that large effects are highly likely for all kinds of zero reflection modes in the system. Phase jumps and singularities make the phase ill-defined, in which case the reflection coefficient absolute value must go to zero for causality reasons. In nonlocal materials such as our TD plasmonic films, phase jumps and singularities come from material spatial dispersion in addition to that of the light beam itself. This is precisely what we study here. The respective extra terms are derived for the structure shown in Figure 1 and can be found in Appendix A. Expressions similar to Eqs. (1) and (2) can be written for *s*-polarized waves as well. However, as they show no peculiarities such as those we are about to discuss, we leave them out (see Appendix A). The derivations of relevance can also be found in Refs. [2], [37].

## 3 Confinement-induced nonlocal electromagnetic response

The electrostatic Coulomb field produced by confined distance-separated charge carriers outside of their confinement region starts playing a perceptible role with confinement size reduction [33], [38]. The Coulomb interaction of charges confined is stronger than that in a homogeneous medium with the same dielectric permittivity constant due to the increased field contribution from outside dielectric environment, given that it has lower dielectric permittivity. That is why to describe the optical properties of TD plasmonic films we use the confinement-induced nonlocal EM response theory built on the Keldysh–Rytova (KR) electron interaction potential [22], [23]. This theory applies nicely to our configuration of an optically dense metallic material slab (plasmonic film) in region 2 of thickness *d* surrounded by semi-infinite dielectrics of constant permittivities *ϵ*_1_ (top) and *ϵ*_3_ (bottom), to result in the in-plane EM response of medium 2 (confined region) as follows

(3) $$\epsilon_{2}\left(\omega ,k\right)=\epsilon_{b}\left[1-\frac{\omega_{p}^{2}\left(k\right)}{\omega \left(\omega +\text{i}\Gamma_{D}\right)}\right].$$

Here, *ϵ*_*b*_ (≫*ϵ*_1_, *ϵ*_3_) is the constant background permittivity of the optically dense plasmonic material of the film, Γ_*D*_ is its damping constant, and its plasma frequency

(4) $$\omega_{p}\left(k\right)=\frac{\omega_{p}^{3\text{D}}}{\sqrt{1+1/\left(\tilde{\epsilon }kd\right)}},\;\;\;\;\;\tilde{\epsilon }=\frac{\epsilon_{b}}{\epsilon_{1}+\epsilon_{3}}$$

is nonlocal (dependent on the in-plane electron momentum *k* = *k*_0_ *n*_1_ sin*ϑ*_*i*_) due to the vertical electron confinement, turning in the limit of *d* → ∞ into $\omega_{p}^{3\text{D}}=\sqrt{4\pi e^{2}N_{3D}/\left(\epsilon_{b}m^{*}\right)}$ of the standard local (Drude) EM response of 3D metals with electron effective mass m* and volumetric electron density *N*_3*D*_ [22]. More precisely, if $\tilde{\epsilon }kd\gg 1$ (relatively thick film), then $\omega_{p}=\omega_{p}^{3D}$ . If $\tilde{\epsilon }kd\ll 1$ (thin enough film), then $\omega_{p}=\sqrt{4\pi e^{2}\tilde{\epsilon }N_{2D}k/\left(\epsilon_{b}m^{*}\right)}$ with *N*_2*D*_ = *N*_3*D*_ *d* being the surface electron density, consistent with plasma frequency of 2D electron gas sandwiched between top (*ϵ*_1_) and bottom (*ϵ*_3_) dielectric materials. Films with $\tilde{\epsilon }kd=\tilde{\epsilon }dk_{0}n_{1}\;\sin\vartheta_{i}≲1$ are referred to as TD films here, so that even relatively thick films can be in the nonlocal TD regime if *ϑ*_*i*_ is small enough.

This theoretical model is verified experimentally [13], [14], [15], [16], which is why we choose to set up

(5) $$\epsilon_{2}\left(\omega ,k\right)=\epsilon_{\text{TiN}}\left(\omega ,k\right)$$

in our numerical studies, with TiN material parameters taken from experimental work [15] and collected in Table 1. The beam waist value is taken from Ref. [37], where it was used to simulate optical reflection processes. Note that Γ_*D*_ starts increasing rapidly for decreasing *d* ≲ 10 nm [15]. However, for the range of *d* used here, it is equal to the bulk value presented in the table.

**Table 1:** Material and Gaussian light beam parameters used.

*ϵ*_*b*_ (TiN)	*ϵ*_1_ (air)	*ϵ*_3_ (MgO)	$\omega_{p}^{3\text{D}}$ (TiN), eV	Γ_D_ (TiN), eV	*w*_0_ (beam waist), μm
9.1	1.0	3.0	2.5	0.2	32

With Eqs. (3)–(5), the derivative *∂ϵ*_2_/*∂k* in the nonlocal reflection coefficient expressions above reads as follows

(6) $$\begin{array}{ll} \frac{\partial \epsilon_{\text{TiN}}\left(\omega ,k\right)}{\partial k} & =-\frac{\epsilon_{b}\omega_{p}^{2}\left(k\right)}{k\left(\tilde{\epsilon }kd+1\right)\omega \left(\omega +\text{i}\Gamma_{D}\right)}\\ & =\frac{\tilde{\epsilon }d\left[\epsilon_{\text{TiN}}\left(\omega ,k\right)-\epsilon_{b}\right]}{{\left(\tilde{\epsilon }kd+1\right)}^{2}}. \end{array}$$

It can be seen not only being nonzero at finite *d* but also being both positive and negative depending on the frequency and direction of the incoming light beam. It disappears for both *d* → 0 and *d* → ∞ as it should to indicate the absence of plasmonic material and to make the EM response of thick plasmonic films local in accord with the standard Drude model, respectively.

## 4 Reflection singularities

For a free standing plasmonic film of thickness *d* in air (Figure 1), the *p*-polarized wave reflection coefficient is [39]

(7) $$R_{p}=\frac{r_{p}^{12}+r_{p}^{23}{\text{e}}^{2\text{i}\gamma_{2}d}}{1+r_{p}^{12}r_{p}^{23}{\text{e}}^{2\text{i}\gamma_{2}d}},$$

with medium 1 (superstrate) and medium 3 (substrate) having the same permittivities *ϵ*_1_ = *ϵ*_3_ = 1. For medium 2 (film), we use *ϵ*_2_ = *ϵ*_TiN_ taking a TiN example of TD material that surpasses noble metals such as Au and Ag [40]. The latter have exceptional plasmonic properties but relatively low melting temperatures making them incompatible with semiconductor fabrication technologies. On the contrary, transition metal nitrides have low-loss plasmonic response, high melting point, and structural stability that makes them capable of forming stoichiometrically perfect TD films down to 1 nm in thickness at room temperature [13], [15]. The Fresnel reflection coefficients $r_{p}^{ij}$ (*i*, *j* = 1, 2, 3) for interfaces between medium 1 and 2 and between medium 2 and 3 are defined as follows

(8) $$r_{p}^{ij}=\frac{\gamma_{i}\epsilon_{j}-\gamma_{j}\epsilon_{i}}{\gamma_{i}\epsilon_{j}+\gamma_{j}\epsilon_{i}},$$

where $\gamma_{i}=\sqrt{k_{0}^{2}\epsilon_{i}-k^{2}}$ are the wave vectors components normal to the interface. Here, $r_{p}^{23}=-r_{p}^{12}$ as *ϵ*_1_ = *ϵ*_3_ = 1, in which case zeroes of *R*_*p*_ are determined by the Brewster mode (BM) condition $r_{p}^{12}=0$ at the film–air interface, whereby *γ*_1_ *ϵ*_2_ = *γ*_2_ *ϵ*_1_, leading to the dispersion relation

(9) $$k=\frac{\omega }{c}\sqrt{\frac{\epsilon_{\text{TiN}}^{\prime }}{\epsilon_{\text{TiN}}^{\prime }+1}}.$$

Also, zeros of *R*_*p*_ can come from the film standing wave (SW) condition, 1 − exp(2i*γ*_2_ *d*) = 0, in which case

(10) $$k=\sqrt{\frac{\omega^{2}}{c^{2}}\epsilon_{\text{TiN}}^{\prime }-{\left(\frac{n\pi }{d}\right)}^{2}}.$$

Here, *n* = 1, 2, 3, … and $\epsilon_{\text{TiN}}^{\prime }=\text{Re}\;\epsilon_{\text{TiN}}$ . Lastly, zero reflection can also occur at the Christiansen point (CP) where $\epsilon_{\text{TiN}}^{\prime }=1$ . If one uses the local Drude model (a “workhorse” routinely used in plasmonics), then *ω*_CP_ = 4 × 10^15^ rad/s comes out of it for any angle of incidence, whereas in the nonlocal KR model used here, the confinement-induced nonlocality of the EM response function *ϵ*_TiN_(*ω*, *k*) makes the CP depend on *k* and thus on the incidence angle.

**Figure 2:**
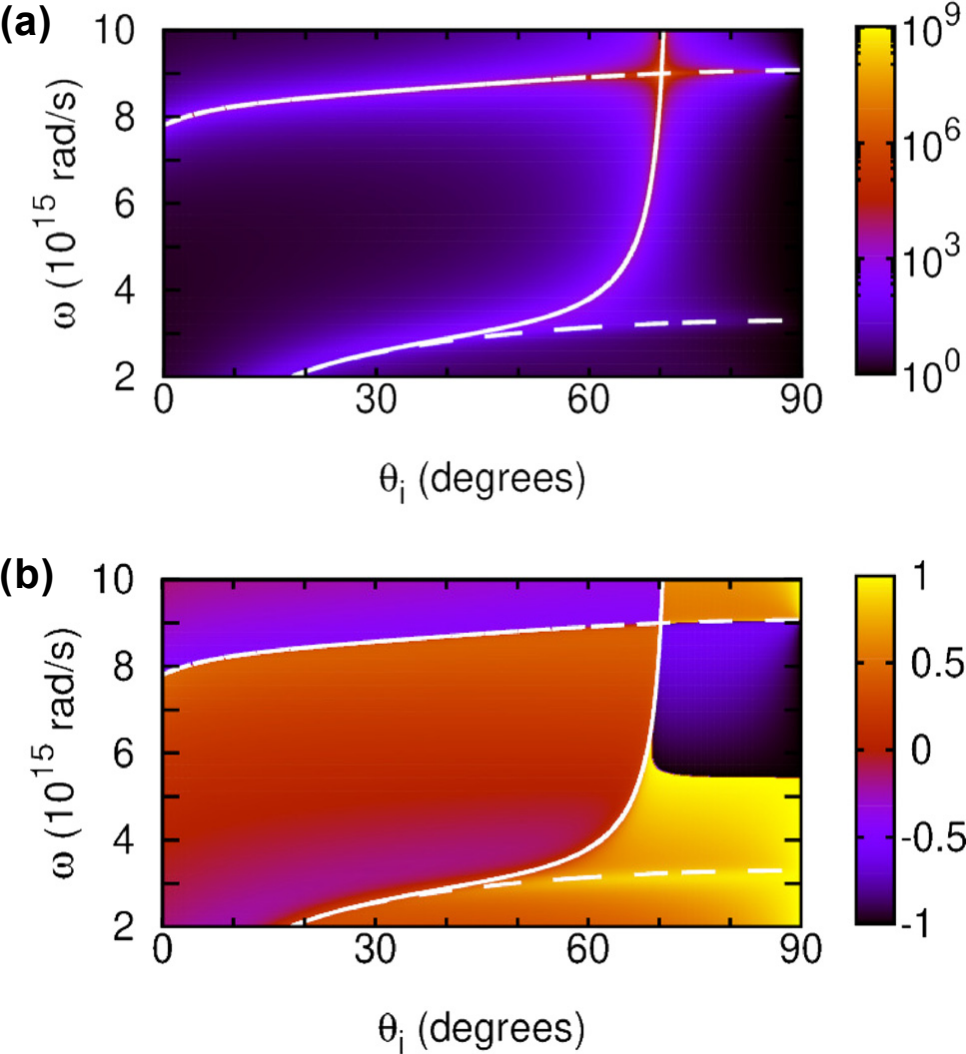
Inverse reflectivity |*R*_*p*_|^−2^ (a) and reflection phase *φ*_*p*_/*π* (b) for the 40 nm thick free-standing nonlocal TiN film. Shown are the (*n* = 1)-SW (upper dashed line), the nonlocal CP (lower dashed line), and the BM (solid line).

**Figure 3:**
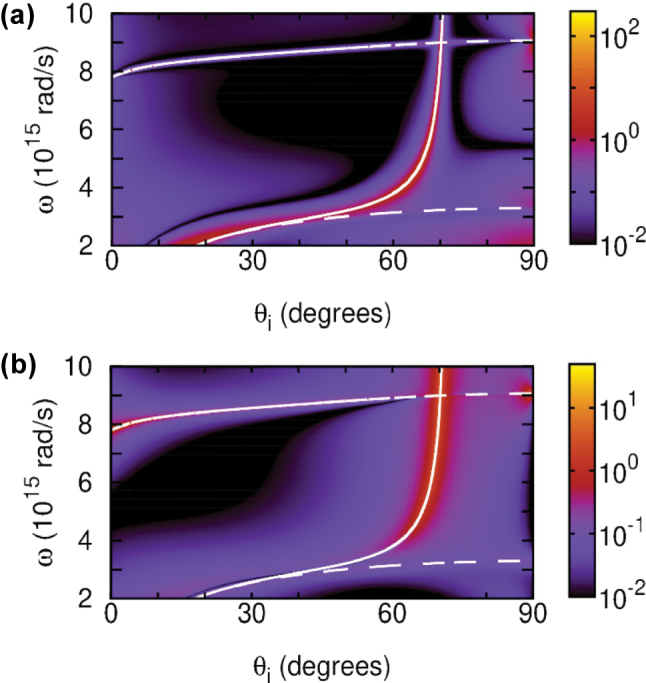
|Δ_GH_| in μm (a) and |Θ_GH_| in mrad (b) calculated for *p*-polarized light wave incident on the 40 nm thick TiN film with inverse reflectivity and reflection phase shown in Figure 2.

Figure 2 shows the inverse reflectivity function 1/|*R*_*p*_|^2^ and reflection phase *φ*_*p*_/*π* calculated from Eqs. (7) and (8) for the 40 nm thick free standing TiN slab with nonlocal EM response. The 40 nm thickness is chosen here and below as an example to show the GH effect enhancement in the visible range; otherwise, it shifts to either IR or UV as can be seen from Eq. (52) (Appendix B.1). Reflectivity reduction and phase jumps can be seen when the SW, BM, or CP mode is excited in the system. The BM still exists below the bulk TiN plasma frequency $\omega_{p}^{3\text{D}}=3.8\times 10^{15}\;rad/s$ , making the GH shifts observable for $\omega <\omega_{p}^{3\text{D}}$ , including the visible range that is impossible to access with local Drude-like plasmonic materials. Figure 3, calculated from Eqs. (1) and (2) for the same example, shows strong lateral and angular GH shifts when the BM is excited, up to ≈80 μm and ≈30mrad , respectively. TD plasmonic materials thereby open access to the GH effect observation with visible light due to their remarkable property of the confinement-induced nonlocal EM response.

**Figure 4:**
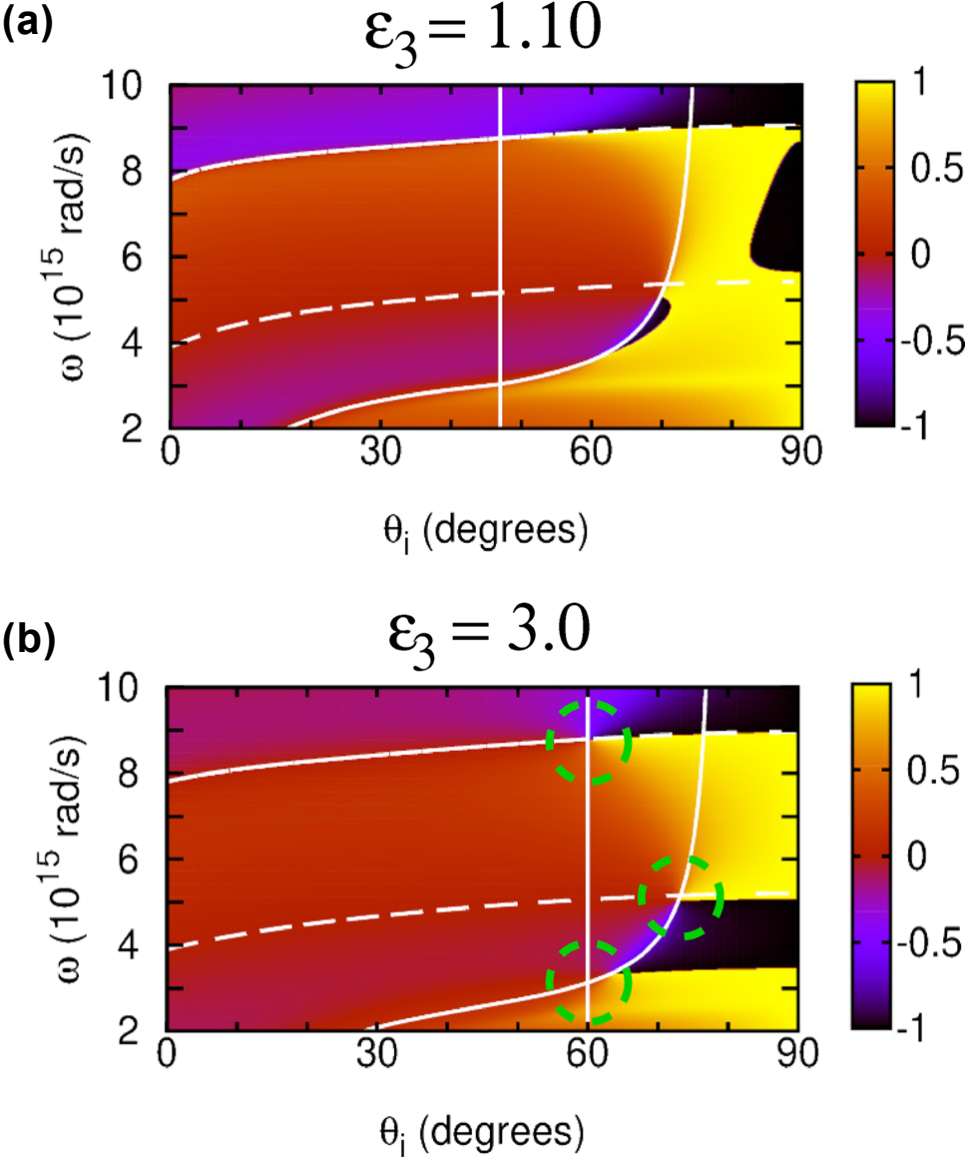
Reflection phase *φ*_*p*_/*π* calculated for a TiN film of *d* = 40 nm with varied *ϵ*_3_ (substrate) and *ϵ*_1_ = 1 (superstrate). Vertical line is the *d* = 0 BM, dashed lines are the first SWs of Eq. (10) with *n* = 0.5, 1, and solid line is the gBM of Eq. (14) (see text). Green circles mark the phase singularity points.

For TD plasmonic films sandwiched between superstrates and substrates with *ϵ*_1_ ≠ *ϵ*_3_, the in-plane reflection symmetry is broken and top-bottom interface mode degeneracy is lifted. Figure 4 shows the birth and development of phase singularities in this case as with *ϵ*_1_ = 1 (air) the substrate permittivity rises up to *ϵ*_3_ = 3 (MgO typically used in TiN thin film systems [15]). It can be seen that just a slight substrate–superstrate dielectric permittivity difference gives birth to the two phase singularities close to the BM phase jump. They have opposite winding numbers (topological charges)

(11) $$C=\frac{1}{2\pi }\oint_{\alpha }\nabla \varphi_{p}\cdot \text{d}s=\pm 1,$$

where *α* is a closed integration path around the phase singularity point. There is also another phase singularity on the SW branch, which moves for larger substrate permittivities close to the SW and BM crossing point. Such singularities result in zero reflection previously reported as “points of topological darkness” for specially designed metasurfaces [41], [42] and multilayer nanostructures [43], [44]. Here, we observe these topologically protected singularity points in mere nonlocal TD films. Remarkably, though, due to the plasma frequency decrease with thickness *d* in our case [15], [22], the gap in Figure 4 between the low-frequency opposite topological charge singularities widens in thinner films (not shown), to red-shift the lower- and blue-shift the higher-frequency singularity points, respectively. By reducing *d* in a controllable way in our case, it can, therefore, be possible to access the low frequency phase singularity point with He-Ne laser at *λ* = 632.8 nm (3 × 10^15^ rad/s) to observe an enhanced GH effect in the visible range. This remarkable feature is only offered by the TD plasmonic films due to their confinement-induced nonlocality and can never be realized with local Drude-like materials.

## 5 Discussion

To understand the origin of the topological singularities discussed, it is instructive to represent the condition *R*_*p*_ = 0 in the form

(12) $$r_{p}^{23}{\text{e}}^{2\text{i}\gamma_{2}d}=-r_{p}^{12}$$

with dissipation temporarily neglected. In this case, the interface reflection coefficients are real numbers for all *ω* where *ϵ*_2_(*ω*, *k*) > 0 (and *γ*_2_ > 0 accordingly) so that our TD film behaves as a dielectric. The LHS of this equation makes a circle in the complex plane of radius $r_{p}^{23}$ with phase *θ* = 2*γ*_2_ *d*, while the RHS is a real number varying between −1 and +1 as *ω* and *θ* change. This is why the equality can only be achieved if the LHS is a real number as well, yielding two cases for Eq. (12) to fulfill. They are (1) ${\text{e}}^{2\text{i}\gamma_{2}d}=+1$ , $r_{p}^{23}=-r_{p}^{12}$ and (2) ${\text{e}}^{2\text{i}\gamma_{2}d}=-1$ , $r_{p}^{23}=r_{p}^{12}$ . Here, the first equations are the SW condition $1-{\text{e}}^{2\text{i}\gamma_{2}d}=0$ and its alternative $1+{\text{e}}^{2\text{i}\gamma_{2}d}=0$ . Both of them lead to the same dispersion relation of Eq. (10), but the former for *n* = 1, 2, 3, … and the latter for *n* = 0.5, 1.5, 2.5, … describing SWs with multiples of a quarter wavelength and so to be referred to as standing quarter waves (SQW), accordingly. The second equation of case 1 is per Eq. (8) fulfilled if

(13) $$\gamma_{1}\epsilon_{3}=\epsilon_{1}\gamma_{3}.$$

This is the substrate–superstrate interface BM condition, which can only be realized hypothetically for *d* = 0, and so to be refereed to as zero-thickness BM (zBM). Similarly, the second equation of case (2) is fulfilled if

(14) $$\gamma_{2}^{2}\epsilon_{1}\epsilon_{3}=\epsilon_{2}^{2}\gamma_{3}\gamma_{1}.$$

For *ϵ*_3_ = *ϵ*_1_, this reduces to the symmetric in-plane interface condition leading to the BM dispersion relation of Eq. (9). However, since it also holds for a broken in-plane reflection symmetry with *ϵ*_3_ ≠ *ϵ*_1_, we refer to the solution of this equation as the generalized BM (gBM).

The two simultaneous equations of cases 1 and 2 are represented by the lines in the 2D configuration space spanned by frequency and angle of incidence. The solutions to Eq. (12) are given by the intersection of these lines. This is where the phase singularity points come from that we obtain at certain frequencies and incident angles. Additionally, there is a singularity coming from the trivial solution of Eq. (12), to give case 3 where $r_{p}^{23}=r_{p}^{12}=0$ , or $r_{p}^{23}=-r_{p}^{12}$ and $r_{p}^{23}=r_{p}^{12}$ simultaneously. This leads to the phase singularity at the intersection of the air–TiN and TiN–MgO interface BMs, or more generally, at the intersection of zBM and gBM lines in the configuration space. A complimentary analysis of the Christiansen points can be found in Appendix B.

**Figure 5:**
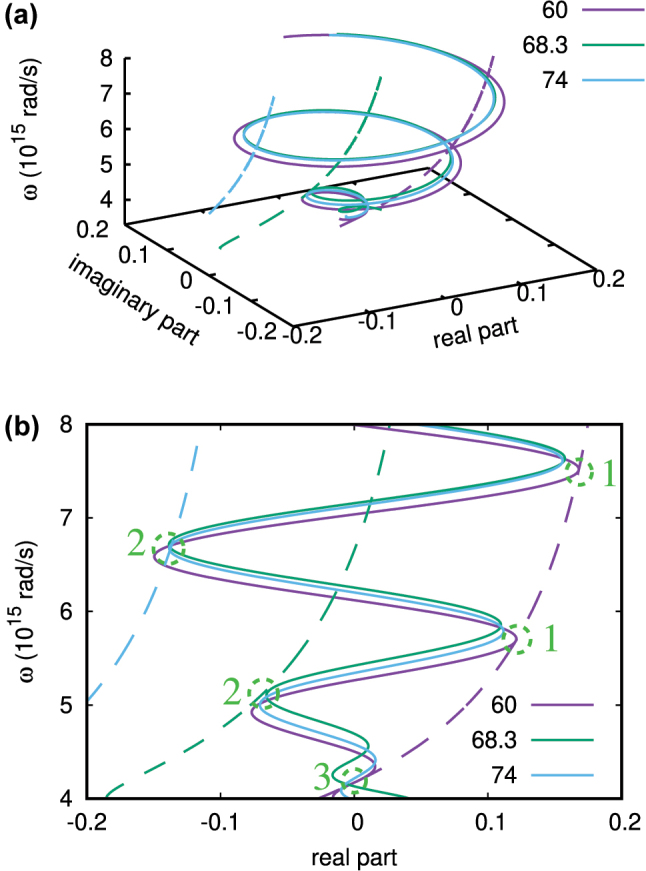
Graphical illustration of the solutions of Eq. (12). (a) Real and imaginary parts of the LHS (solid lines) and RHS (dashed lines) of Eq. (12) for a few incidence angles. (b) Projection of (a) on the plane spanned by *ω* and real part. Angles are chosen to show the three solution cases to yield phase singularities in an air/TiN/MgO structure with *d* = 150 nm. Green circles mark the phase singularity points.

Figure 5(a) shows the real and imaginary parts of Eq. (12) calculated for a thicker TiN film of *d* = 150 nm (to include more solution points) as functions of frequency for a few incident angles fixed. In Figure 5(b) that presents a projection of (a), solution cases 1, 2, and 3 discussed above are marked accordingly. Here, case 1 can be seen to generate an infinite number of discrete solution points for a single 60° incident angle as *ϵ*_1_ and *ϵ*_3_ in Eq. (13) are frequency independent constants. Case 2 yields the solution points at different incident angles 68.3°, 74°, 76° (not shown), etc. (also an infinite number, in principle) due to the strong gBM frequency dependence in Eq. (14). The case 3 solution point can be seen at the intersection of the two lines of the 60° incidence angle, to yield the phase singularity point that can be shifted down to the visible *ω* for thinner films (see below) due to the confinement-induced nonlocal EM response.

**Figure 6:**
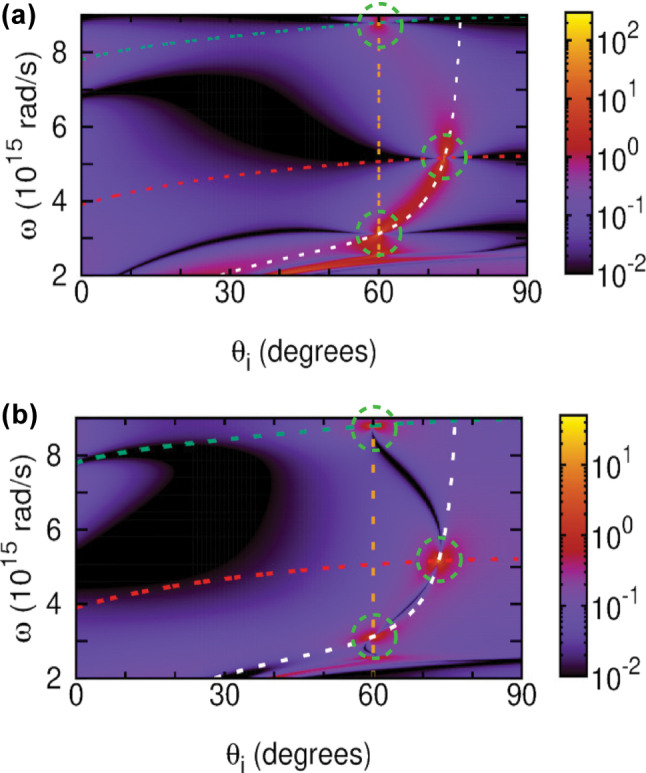
Absolute values of lateral (a) and angular (b) GH shifts |Δ_GH_| (μm) and |Θ_GH_| (mrad) calculated neglecting dissipation for the air/TiN/MgO system with 40 nm thick TiN film. Green (red) dashed line is the SW (SQW) with *n* = 1 (*n* = 0.5) of Eq. (10). Vertical orange dashed line is the zBM of Eq. (13). White dashed line is the gBM from Eq. (14). Circles indicate the three cases of phase singularity points.

**Figure 7:**
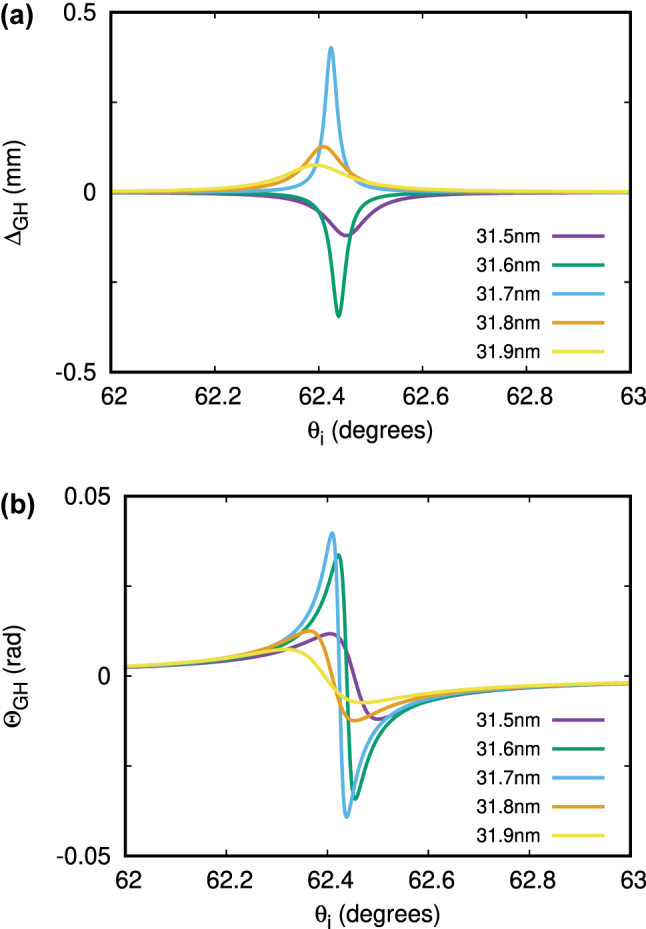
Lateral (a) and angular (b) GH shifts Δ_GH_ (mm) and Θ_GH_ (rad) calculated for the nonlocal dissipative air/TiN/MgO system with TiN thickness in the vicinity of *d* = 31.7 nm and *θ*_*i*_ = 62.42° of the He-Ne laser beam (case 3 phase singularity point).

The impact of phase singularities on the GH shifts is shown in Figure 6 for the air/TiN/MgO system of the 40 nm thick TiN film with dissipation neglected. Comparing to Figure 4(b), the GH shifts can be seen to be very pronounced at the phase singularity points, which move with dissipation just slightly (not shown), for all three cases discussed. Using thinner TD films with broken in-plane symmetry, it is even possible to make these GH shift singularities observable in the visible range under He-Ne laser excitation (*ω* = 3 × 10^15^ rad/s). Figure 7 shows the GH shifts under such excitation with angle of incidence varied around *θ*_*i*_ = 62.42° for the same air/TiN/MgO system (dissipation included) with TiN film thickness varied around *d* = 31.7 nm to encounter the case 3 phase singularity point. For *d* slightly thicker or thinner than that the singularity shows up slightly red- or blue-shifted, depending on its topological charge, making Δ_GH_ of Eq. (1) change its sign as *d* decreases. Similarly, the sign of Θ_GH_ of Eq. (2) changes as the incidence angle varies around *θ*_*i*_ = 62.42° of the case 3 phase singularity point. Most important though is that both lateral and angular GH shifts shown are extremely large, in the millimeter and a few tens of milliradian range, respectively, over an order of magnitude greater than those shown in Figure 3 for a similar in-plane symmetric TD film system.

## 6 Conclusions

To summarize, we show that the TD plasmonic films with broken in-plane reflection symmetry can feature an extraordinarily large GH effect in the visible range due to the topologically protected phase singularities of their reflection coefficient. We classify the phase singularities and provide a detailed analysis for the conditions under which they emerge in the simplest possible system of a single TD plasmonic film (TiN) deposited on a low-permittivity dielectric substrate (MgO) in air. We emphasize that the existence of a phase singularity for a single plasmonic film in the visible stems from the confinement-induced EM response nonlocality (in-plane momentum dependence) of the system, with plasma frequency decreasing as the film gets thinner [15]. We note also that this confinement-induced nonlocality comes as a product of thickness by the in-plane momentum. So, the nonlocal plasma frequency can be red-shifted (and thus brought to the visible) by simultaneous reduction of thickness and in-plane momentum through the incident angle change of the incoming light beam. It is this that provides such a remarkable opportunity to have the strong GH effect in the visible range for not very thin (31–32 nm, Figure 7) TiN films, which can be routinely fabricated in the lab and are considered here theoretically as a demonstrative example. This is not possible for conventional (about an order of magnitude thicker) thin metallic films due to their local EM response with plasma frequency being a constant in the near-UV.

Previous studies reported an overall GH shift of 325 μm by coupling incident light to a surface plasmon polariton of a plasmonic film through the use of a prism [36], as well as lateral and angular GH shifts as large as 70 times 785 nm incident wavelength and 200 μrad, respectively, for artificially designed hybrid multilayer metasurface structures [45]. In contrast, our analysis here reveals even greater lateral and angular GH shifts ∼0.4 mm (632 times the wavelength) and ∼40 mrad for visible He-Ne laser light with simple TD plasmonic films, where Nature itself does the job to greatly enhance the GH effect, indicating that such systems could provide a new flexible quantum material platform to offer new opportunities for quantum optics, quantum computing, and biosensing application development.

## Figures and Tables

**Figure 8:**
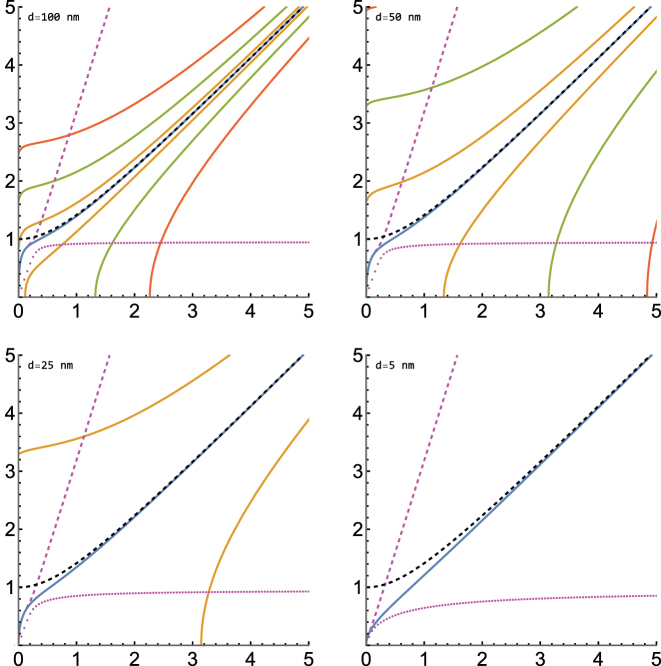
Propagating and evanescent standing wave solutions with *n* = 0, 1, 2, and 3 calculated for the free standing TiN films of decreasing thickness *d* from Eq. (52) with frequency *ω* in units of $\omega_{p}^{3\text{D}}$ and in-plane momentum *k* in units of $\sqrt{\epsilon_{b}}\;\omega_{p}^{3\text{D}}/c$ . In a relatively thick film (*d* = 100 nm), the *n* ≠ 0-modes can be seen to group around the fundamental plasma mode (*n* = 0, shown by blue and black dashed line for nonlocal KR and local Drude in-plane EM response given by Eqs. (3) and (4) for finite and infinitely large *d*, respectively); propagating modes are above and evanescent modes are below the *n* = 0-mode. In the limit *d* → ∞, all of them congregate together to form the multiply degenerate bulk plasma mode with standard low-*k* dispersion $\omega ∼\omega_{p}^{3\text{D}}$ . As *d* decreases, all *n* ≠ 0-modes spread out to reflect the strengthening of the vertical confinement in the TD film system. In ultrathin films (*d* = 5 nm), only the fundamental *n* = 0-mode remains with low-*k* dispersion distinctly different for nonlocal and local in-plane EM response of the film ( $\omega ∼\sqrt{k}$ versus $\omega ∼\omega_{p}^{3\text{D}}$ ) [22], [26]. Purple dashed and dotted lines indicate the Brewster and surface modes, respectively, of Eq. (45).

**Figure 9:**
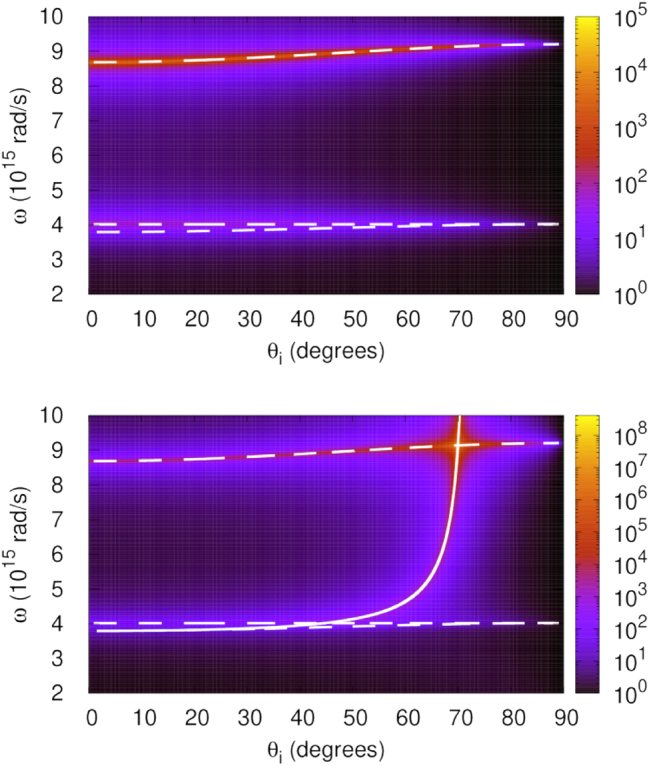
Inverse reflectivity |*R*_*s*/*p*_|^−2^ for *s*-polarized (top panel) and *p*-polarized (bottom panel) light calculated for a 40 nm thick TiN film using the local (Drude) in-plane EM response function one can obtain from Eqs. (3) and (4) in the limit *d* → ∞. The white solid line is the Brewster mode of Eq. (45); it can be seen to only exist for *p*-polarized light (bottom panel). The horizontal dashed line at *ω* = 4 × 10^15^ rad/s indicates the Christiansen point (see the text below). Two other dashed lines shown are tilted, depend on the angle of incidence. They are the *n* = 0 fundamental plasma mode (lower dashed line) and the *n* = 1 standing wave mode (upper dashed line), both given by Eq. (47). For all modes mentioned, the reflectivity can be seen to be close to zero for both *s*- and *p*-polarized light.

**Figure 10:**
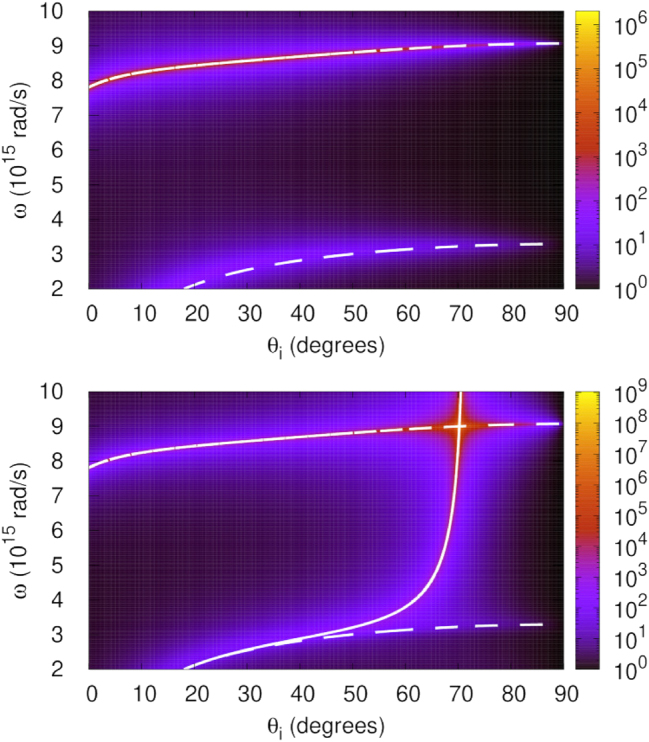
Same as in Figure 9 but now for a 40 nm thick TiN film described by the nonlocal KR in-plane EM response function given by Eqs. (3) and (4). Shown are the *n* = 0 fundamental plasma mode (lower dashed curve) and the *n* = 1 standing wave (upper dashed curve) of Eq. (47) as well as the Brewster mode of Eq. (45) (solid line for *p*-polarization). The behavior of all modes can be seen to drastically change for small angles of incidence (cf. also Figure 8).

**Figure 11:**
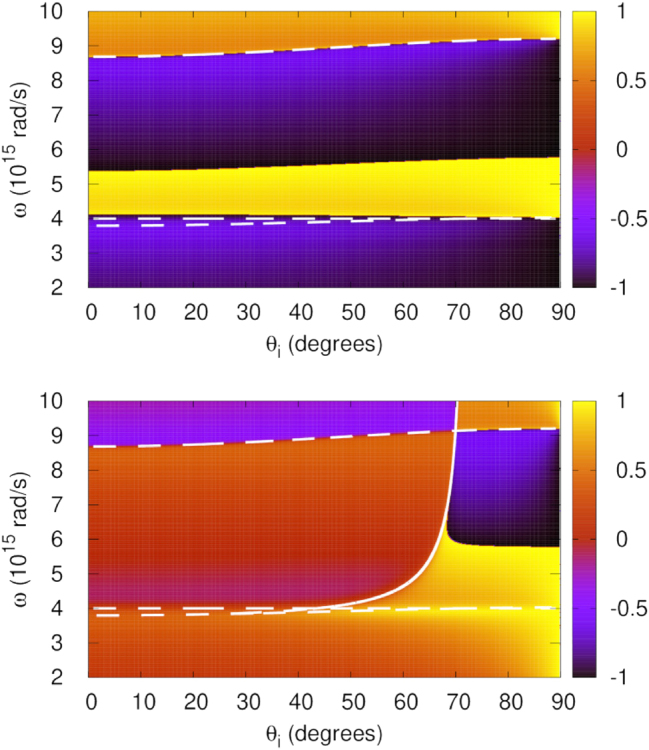
Phase (normalized by *π*) for the reflection coefficients calculated using the local (Drude) in-plane EM response function, whose inverse squares are shown in Figure 9. The dashed and solid lines are the same as in Figure 9. The phase jumps can be seen at the *n* = 1 standing wave mode (upper dashed line) for both *s*- and *p*-polarizations. Extra phase jumps occur between the dashed lines and close to the Brewster mode (solid line) for *s*- and *p*-polarizations, respectively.

**Figure 12:**
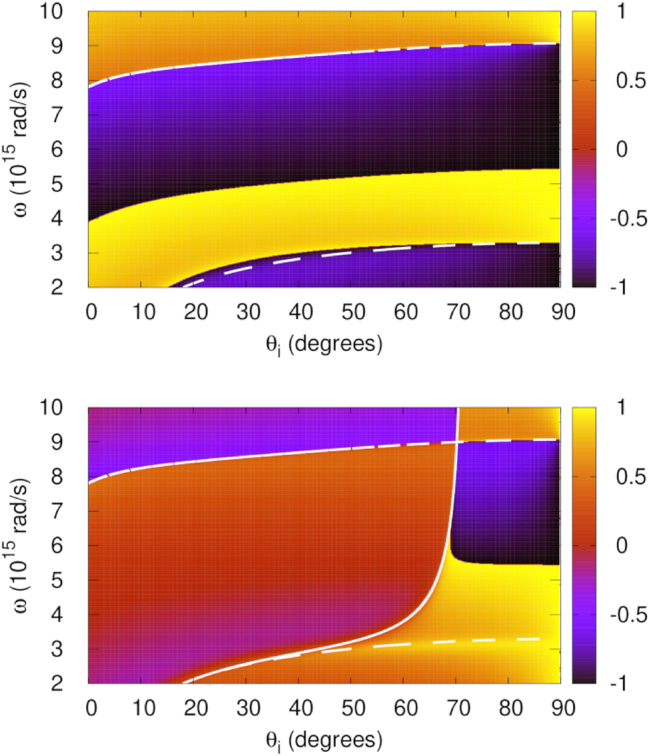
Phase (normalized by *π*) for the reflection coefficients described by the nonlocal KR in-plane EM response function, whose inverse squares are shown in Figure 10. The dashed and solid lines are the same as in Figure 3. The impact of the EM response nonlocality can be seen at small angles of incidence.

**Figure 13:**
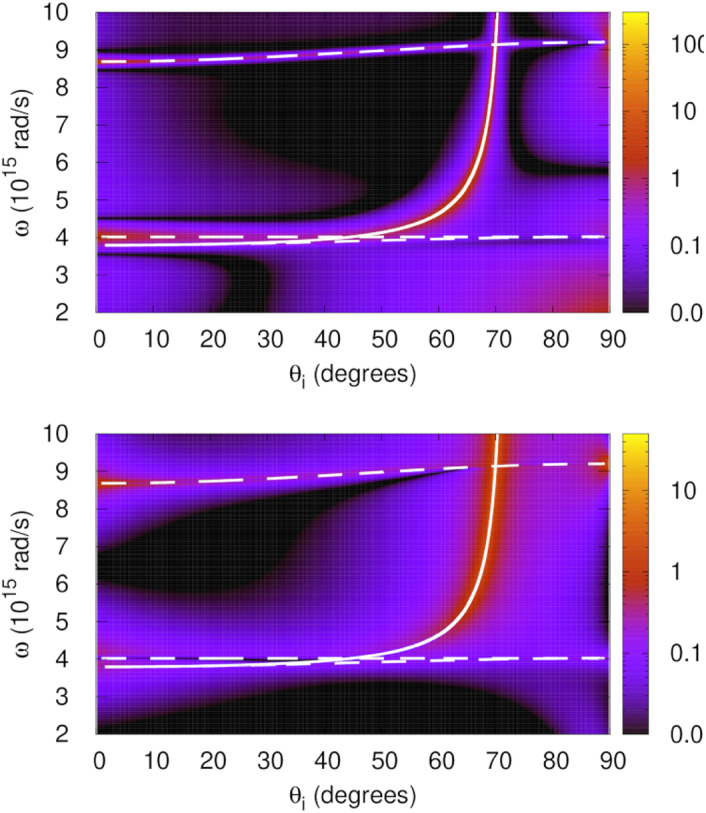
GH shifts Δ_GH_ in μm (top) and Θ_GH_ in mrad (bottom) for *p*-polarized light impinging on a 40 nm thick *local* TiN film with inverse reflectivity and phase shown in Figures 9 and 11, respectively. The solid and dashed lines are the same as in the bottom panel of Figure 9. It can be seen that the lateral shift Δ_GH_ (top) is particularly large for the Brewster mode (solid line) and the standing wave solutions with *n* = 0 (fundamental plasma mode) and *n* = 1, as well as for the Christiansen point (at small angels of incidence). The angular shift Θ_GH_ (bottom) is particularly large when the Brewster mode condition (solid line) and the *n* = 1 standing wave condition (upper dashed line) are fulfilled.

**Figure 14:**
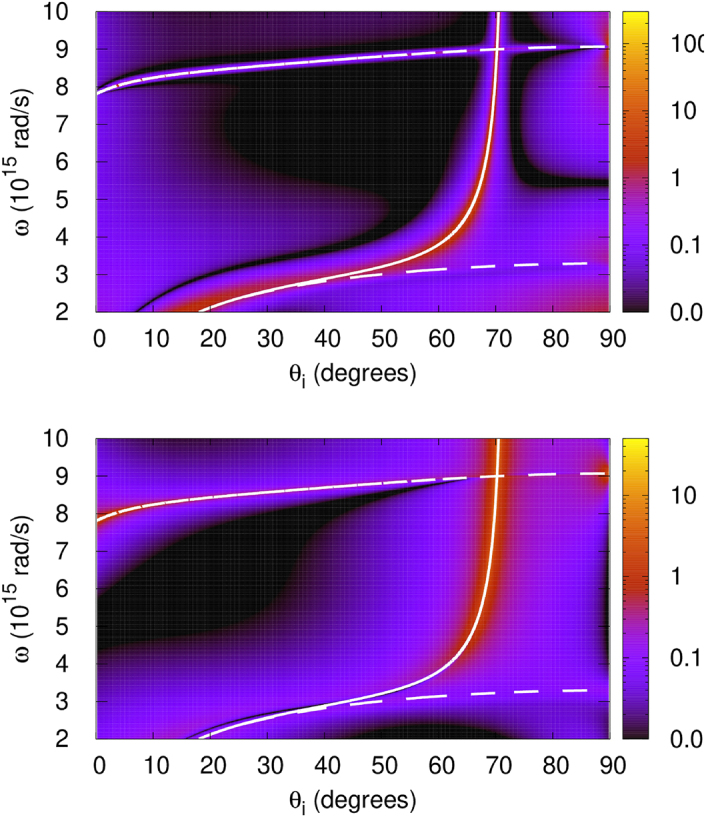
Same as in Figure 13 for *p*-polarized light impinging on a 40 nm thick *nonlocal* TiN film with inverse reflectivity and phase shown in Figures 10 and 12, respectively. The lines are the same as in Figure 10. The impact of the EM response nonlocality can be seen at small angles of incidence.

**Figure 15:**
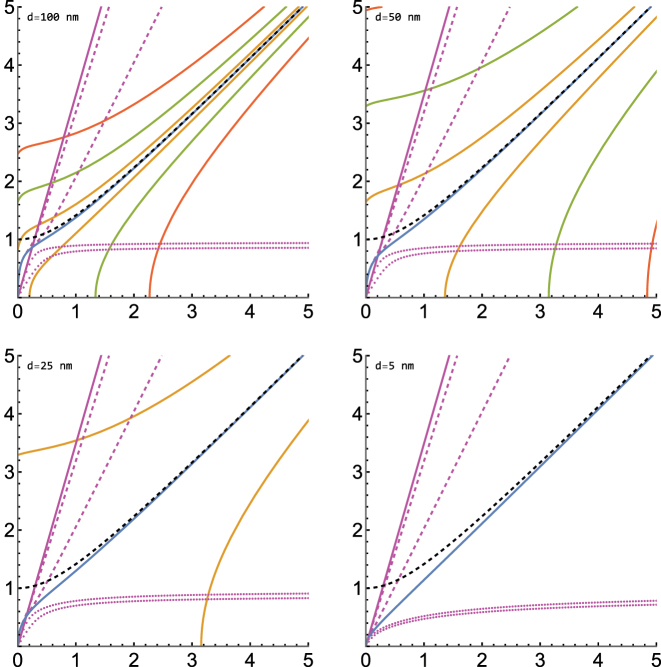
Same as in Figure 8 for air/TiN/MgO TD films. Purple dashed and dotted lines show the split-up nondegenerate Brewster and surface modes, respectively, given by Eq. (45) for the air/TiN interface and by Eq. (53) for the TiN/MgO interface. Purple solid line is the Brewster mode of the hypothetical air/MgO interface introduced in (4.), discussed in (A)–(C), and referred to as zero Brewster mode (zBM) in the main text. The zBM intersection points with standing wave modes (including the *n* = 0 fundamental plasma mode) are the phase singularity points (or points of topological darkness) to yield *R*_*p*_ = 0 and thus to greatly enhance the GH effect.

**Figure 16:**
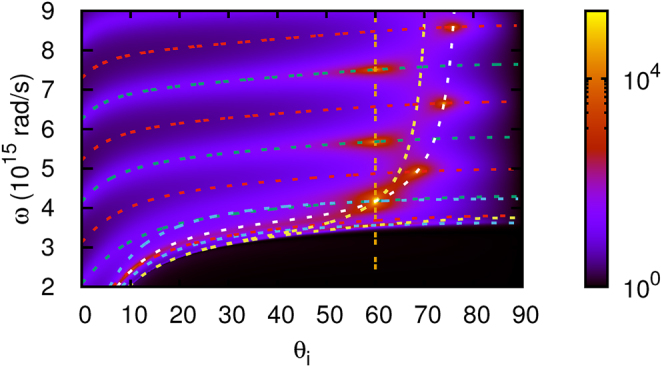
Inverse reflectivity 1/|*R*_*p*_|^2^ for a 150 nm thick TiN film on MgO calculated using the nonlocal KR in-plane EM response as given by Eqs. (3) and (4) with losses neglected by setting Γ_*D*_ = 0. The figure presents the features listed in the text. They are as follows: (1.) the air/TiN interface Brewster mode is shown by the (upper) yellow dashed line; (2.) the MgO/TiN interface Brewster mode is shown by the (lower) yellow dashed line; (3.) the standing wave solutions for *n* = 1, 2, 3 from Eq. (47) are shown by the green dashed lines; (4.) the vertical dashed orange line indicates the hypothetical MgO/Air interface Brewster mode (also called zBM in the main text); (5.) and (6.) the blue dashed lines mark the two Christiansen points. Additionally presented are the generalized Brewster mode (gBM) from (F) by the white dashed line and the standing wave solutions with *n* = 0.5, 1.5, 2.5, 3.5 from Eq. (47), or the condition in (F), by the red dashed lines. The reflection zeros can be seen at the crossing points of the standing waves from (3.) (green dashed lines) and the zBM from (4.) (vertical dashed line), as described in (B) in the text. More reflection zeroes can be seen at the intersection of the standing wave solutions from (F) (red dashed lines) and the gBM from (F). All the Brewster modes can be seen to intersect with one of the Christiansen points and the *n* = 1 standing wave mode. There is also the intersection of the Brewster modes (2.), (3.) with the Christiansen point at the 60° incidence angle and *ω* = 3.55 × 10^15^ rad/s, to provide another reflection zero (barely seen) corresponding to (A) and (D) listed in the text. All these intersection points provide the phase singularities to enhance the GH shifts.

**Figure 17:**
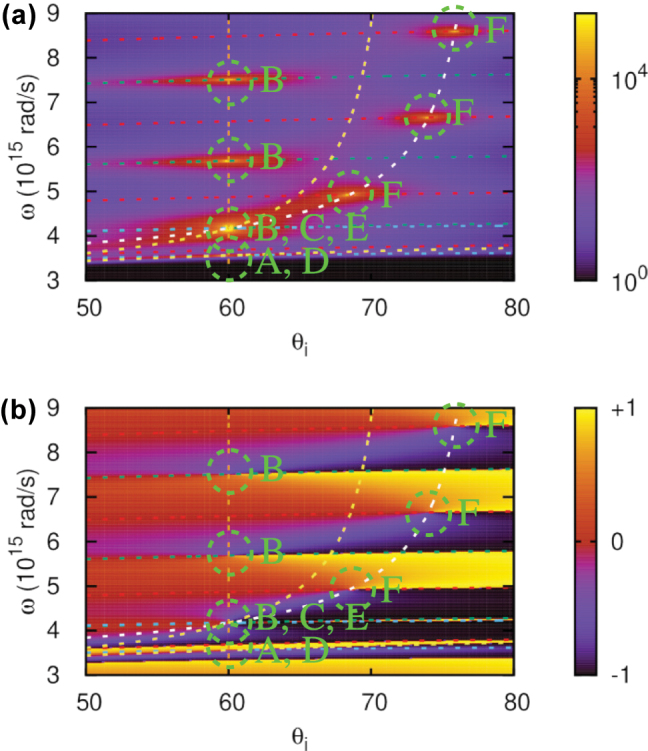
Singularities of the inverse reflectivity to contribute to the GH effect enhancement. (a) Inverse reflectivity 1/|*R*_*p*_|^2^ as in Figure 16 for a 150 nm thick TiN film on MgO and (b) the phase *π*-normalized of the respective reflection coefficient – both graphs are zoomed in to include a smaller range of angles of incidence. The lines are the same as in Figure 16. The labeled green circles mark the mode intersection points and the phase singularities as per the classification (A)–(F) in the text, to enhance the respective GH shifts.

## Appendix A: GH shifts for Gaussian light beams

As it is generally accepted in the literature [46], [47], we assume incoming light to impinge from medium 1 (refractive index *n*_1_) on the surface of medium 2 in the form of an incident *p*-polarized Gaussian beam with the electric field vector component as follows (*k*_0_ = *ω*/*c*)

(15)
$$E_{p}^{\text{inc}}\left(x_{i},y_{i},z_{i}\right)\propto {\text{e}}^{\text{i}k_{0}n_{1}z_{i}-k_{0}n_{1}\frac{x_{i}^{2}+y_{i}^{2}}{2\left(L+\text{i}z_{i}\right)}}\left({\hat{x}}_{i}-\text{i}{\hat{z}}_{i}\frac{x_{i}}{L+\text{i}z_{i}}\right).$$

Here, (*x*_*i*_, *y*_*i*_, *z*_*i*_) are the coordinates in the Cartesian reference frame formed by the orthonormal basis vector set $\left({\hat{x}}_{i},{\hat{y}}_{i},{\hat{z}}_{i}\right)$ attached to the center of the beam cross section such that ${\hat{z}}_{i}$ sets up its propagation direction and ${\hat{x}}_{i}$ lies in the plane of incidence pointing in the direction off the surface, $L=k_{0}w_{0}^{2}/2$ with *w*_0_ representing the beam waist. In full analogy, by implementing the boundary conditions, a similar expression can be written down for $E_{p}^{\text{refl}}$ in the $\left({\hat{x}}_{r},{\hat{y}}_{r},{\hat{z}}_{r}\right)$ reference frame attached to the center of the cross section of the reflected beam. The total GH shift can then be calculated as the mean value

(16)
$$\left\langle x_{r}\right\rangle =\frac{\int \text{d}x_{r}\int \text{d}y_{r}\;I\left(x_{r},y_{r},z_{r}\right)x_{r}}{\int \text{d}x_{r}\int \text{d}y_{r}\;I\left(x_{r},y_{r},z_{r}\right)}$$

of the centroid displacement for the reflected beam [47], where $I\left(x_{r},y_{r},z_{r}\right)\propto |E_{p}^{\text{refl}}\left(x_{r},y_{r},z_{r}\right){|}^{2}$ is the reflected beam intensity in the far-field regime. This yields

(17)
$$\left\langle x_{r}\right\rangle =\frac{1}{k_{0}}\text{Im}\frac{\partial \ln R_{\text{p}}}{\partial \theta_{i}}-\frac{z_{r}}{k_{0}L}\text{Re}\frac{\partial \ln R_{\text{p}}}{\partial \theta_{i}}$$

with *R*_*p*_ representing the Fresnel reflection coefficient for *p*-polarized light, whereby for a detector placed a distance *z*_*r*_ = *l* above the interface one obtains the total GH shift as a sum of the lateral Δ_GH_ and angular Θ_GH_ shifts of the form

(18)
$$\Delta_{\text{total}}=\Delta_{\text{GH}}+l\tan\left(\Theta_{\text{GH}}\right)\approx \Delta_{\text{GH}}+l\Theta_{\text{GH}},$$

(19)
$$\begin{array}{c} \Delta_{\text{GH}}=n_{1}\cos\left(\theta_{i}\right)\;\text{Im}\left[\frac{1}{R_{\text{p}}}\frac{\partial R_{\text{p}}}{\partial k}\right],\\ \Theta_{\text{GH}}=-\frac{\theta_{0}^{2}}{2}k_{0}n_{1}\cos\left(\theta_{i}\right)\;\text{Re}\left[\frac{1}{R_{\text{p}}}\frac{\partial R_{\text{p}}}{\partial k}\right]. \end{array}$$

Here, *θ*_0_ = *λ*/(*πw*_0_) = 2/(*w*_0_ *k*_0_ *n*_1_) and the partial derivatives over *θ*_*i*_ are replaced by those over *k* = *k*_0_ *n*_1_sin(*θ*_*i*_). An in-depth analysis of the GH expressions can be found in Refs. [2], [37].

For a material with a local (*k*-independent) EM response, the derivatives over *k* in Eq. (19) can only be nonzero due to the cross-sectional inhomogeneity of the incoming light beam as can be seen from Eqs. (15)–(17) and the general structure of the *p*-wave reflection coefficient [39],

(20)
$$\begin{array}{c} R_{p}=\frac{r_{p}^{12}+r_{p}^{23}{\text{e}}^{2\text{i}\gamma_{2}d}}{1+r_{p}^{12}r_{p}^{23}{\text{e}}^{2\text{i}\gamma_{2}d}},\;\;\;\;\;r_{p}^{ij}=\frac{\gamma_{i}\epsilon_{j}-\gamma_{j}\epsilon_{i}}{\gamma_{i}\epsilon_{j}+\gamma_{j}\epsilon_{i}},\\ \gamma_{i}=\sqrt{k_{0}^{2}\epsilon_{i}-k^{2}} \end{array}$$

(*i*, *j* = 1, 2, 3), written for a typical case of a finite-thickness material slab of thickness *d* with local EM response *ϵ*_2_ = *ϵ*_2_(*ω*) sandwiched between semi-infinite superstrate and substrate dielectrics of constant permittivities *ϵ*_1_ and *ϵ*_3_, respectively. The *s*-wave reflection coefficient *R*_*s*_ can be obtained by replacing $r_{p}^{ij}$ with $r_{s}^{ij}=\left(\gamma_{i}-\gamma_{j}\right)/\left(\gamma_{i}+\gamma_{j}\right)$ in the above equations. Thus, in Eq. (19), one has

(21)
$$\begin{array}{c} \Delta_{\text{GH}}=\Delta_{\text{GH}}^{\text{loc}}=n_{1}\cos\left(\theta_{i}\right)\;\text{Im}\left[\frac{1}{R_{\text{p}}}{\left(\frac{\partial R_{\text{p}}}{\partial k}\right)}_{\text{loc}}\right],\\ \Theta_{\text{GH}}=\Theta_{\text{GH}}^{\text{loc}}=-\frac{\theta_{0}^{2}}{2}k_{0}n_{1}\cos\left(\theta_{i}\right)\;\text{Re}\left[\frac{1}{R_{\text{p}}}{\left(\frac{\partial R_{\text{p}}}{\partial k}\right)}_{\text{loc}}\right]. \end{array}$$

For a nonlocal material *ϵ*_2_ = *ϵ*_2_(*ω*, *k*), and then there are extra contributions to add to the above, those proportional to *∂ϵ*_2_(*ω*, *k*)/*∂k*, whereby Eq. (19) takes the form

(22)
$$\Delta_{\text{GH}}=\Delta_{\text{GH}}^{\text{loc}}+\Delta_{\text{GH}}^{\text{nloc}}\left(k\right),\;\;\;\;\;\Theta_{\text{GH}}=\Theta_{\text{GH}}^{\text{loc}}+\Theta_{\text{GH}}^{\text{nloc}}\left(k\right)$$

with additional nonlocal terms

(23)
$$\begin{array}{c} \Delta_{\text{GH}}^{\text{nloc}}\left(k\right)=n_{1}\cos\left(\theta_{i}\right)\;\text{Im}\left[\frac{1}{R_{\text{p}}}{\left(\frac{\partial R_{\text{p}}}{\partial k}\right)}_{\text{nloc}}\right],\\ \Theta_{\text{GH}}^{\text{nloc}}\left(k\right)=-\frac{\theta_{0}^{2}}{2}k_{0}n_{1}\cos\left(\theta_{i}\right)\;\text{Re}\left[\frac{1}{R_{\text{p}}}{\left(\frac{\partial R_{\text{p}}}{\partial k}\right)}_{\text{nloc}}\right], \end{array}$$

which do not appear when the material-induced nonlocality is neglected.

In order to calculate the GH shifts in Eq. (22), the partial derivative of the reflection coefficient *R*_*p*_ over *k* should be obtained first. With its definition given by Eq. (20), following is the full list of equations we used to calculate *∂R*_*p*_/*∂k* with both local and nonlocal terms included.

(24)
$$\frac{\partial r_{p}^{ij}}{\partial k}={\left(\frac{\partial r_{p}^{ij}}{\partial k}\right)}_{\text{loc}}+{\left(\frac{\partial r_{p}^{ij}}{\partial k}\right)}_{\text{nloc}}$$

with

(25)
$$\begin{array}{c} {\left(\frac{\partial r_{p}^{ij}}{\partial k}\right)}_{\text{loc}}=-\frac{A_{ij}}{B_{ij}^{2}}B_{ij,loc}^{\prime }+\frac{1}{B_{ij}}A_{ij,loc}^{\prime },\\ {\left(\frac{\partial r_{p}^{ij}}{\partial k}\right)}_{\text{nloc}}=-\frac{A_{ij}}{B_{ij}^{2}}B_{ij,nloc}^{\prime }+\frac{1}{B_{ij}}A_{ij,nloc}^{\prime }, \end{array}$$

where

(26)
$$r_{p}^{ij}=\frac{A_{ij}}{B_{ij}},\;\;\;\;\;A_{ij}=\gamma_{i}\epsilon_{j}-\gamma_{j}\epsilon_{i},\;\;\;\;\;B_{ij}=\gamma_{i}\epsilon_{j}+\gamma_{j}\epsilon_{i},$$

(27)
$$\begin{array}{c} A_{ij,loc}^{\prime }=k\left(\frac{\epsilon_{i}}{\gamma_{j}}-\frac{\epsilon_{j}}{\gamma_{i}}\right),\\ A_{ij,nloc}^{\prime }=\frac{\partial \epsilon_{i}}{\partial k}\left(\frac{\epsilon_{j}k_{0}^{2}}{2\gamma_{i}}-\gamma_{j}\right)-\frac{\partial \epsilon_{j}}{\partial k}\left(\frac{\epsilon_{i}k_{0}^{2}}{2\gamma_{j}}-\gamma_{i}\right), \end{array}$$

(28)
$$\begin{array}{c} B_{ij,loc}^{\prime }=-k\left(\frac{\epsilon_{i}}{\gamma_{j}}+\frac{\epsilon_{j}}{\gamma_{i}}\right),\\ B_{ij,nloc}^{\prime }=\frac{\partial \epsilon_{i}}{\partial k}\left(\frac{\epsilon_{j}k_{0}^{2}}{2\gamma_{i}}+\gamma_{j}\right)+\frac{\partial \epsilon_{j}}{\partial k}\left(\frac{\epsilon_{i}k_{0}^{2}}{2\gamma_{j}}+\gamma_{i}\right). \end{array}$$

The nonlocal term in Eq. (24) can be seen to be proportional to *∂ϵ*_*i*_/*∂k* which in our configuration comes from the nonlocal EM response *ϵ*_*i*=2_ = *ϵ*_2_(*ω*, *k*) of medium 2 situated in between dielectric media with constant *ϵ*_1_ (superstrate) and *ϵ*_3_ (substrate). In view of this, the *∂R*_*p*_/*∂k* derivative splits into the local and nonlocal contributions as follows

(29)
$$\frac{\partial R_{p}}{\partial k}={\left(\frac{\partial R_{p}}{\partial k}\right)}_{\text{loc}}+{\left(\frac{\partial R_{p}}{\partial k}\right)}_{\text{nloc}}$$

with

(30)
$$\begin{array}{c} {\left(\frac{\partial R_{p}}{\partial k}\right)}_{\text{loc}}=-\frac{C}{D^{2}}D_{\text{loc}}^{\prime }+\frac{1}{D}C_{\text{loc}}^{\prime },\\ {\left(\frac{\partial R_{p}^{ij}}{\partial k}\right)}_{\text{nloc}}=-\frac{C}{D^{2}}D_{\text{nloc}}^{\prime }+\frac{1}{D}C_{\text{nloc}}^{\prime }, \end{array}$$

where

(31)
$$R_{p}=\frac{C}{D},\;\;\;\;\;C=r_{p}^{12}+r_{p}^{23}{\text{e}}^{2\text{i}\gamma_{2}d},\;\;\;\;\;D=1+r_{p}^{12}r_{p}^{23}{\text{e}}^{2\text{i}\gamma_{2}d}$$

and

(32)
$$\begin{array}{ll} C_{\text{loc}}^{\prime } & ={\left(r_{p}^{12}\right)}_{\text{loc}}^{\prime }+{\left(r_{p}^{23}\right)}_{\text{loc}}^{\prime }{\text{e}}^{2\text{i}\gamma_{2}d}-r_{p}^{23}{\text{e}}^{2\text{i}\gamma_{2}d}\frac{2\text{i}kd}{\gamma_{2}},\\ C_{\text{nloc}}^{\prime } & ={\left(r_{p}^{12}\right)}_{\text{nloc}}^{\prime }+{\left(r_{p}^{23}\right)}_{\text{nloc}}^{\prime }{\text{e}}^{2\text{i}\gamma_{2}d}+r_{p}^{23}{\text{e}}^{2\text{i}\gamma_{2}d}\frac{\text{i}k_{0}^{2}d}{\gamma_{2}}\frac{\partial \epsilon_{2}}{\partial k}, \end{array}$$

(33)
$$\begin{array}{ll} D_{\text{loc}}^{\prime } & =\left[{\left(r_{p}^{12}\right)}_{\text{loc}}^{\prime }r_{p}^{23}+r_{p}^{12}{\left(r_{p}^{23}\right)}_{\text{loc}}^{\prime }-r_{p}^{12}r_{p}^{23}\frac{2\text{i}k}{\gamma_{2}}\right]{\text{e}}^{2\text{i}\gamma_{2}d},\\ D_{\text{nloc}}^{\prime } & =\left[{\left(r_{p}^{12}\right)}_{\text{nloc}}^{\prime }r_{p}^{23}+r_{p}^{12}{\left(r_{p}^{23}\right)}_{\text{nloc}}^{\prime }-r_{p}^{12}r_{p}^{23}\frac{\text{i}k_{0}^{2}}{\gamma_{2}}\frac{\partial \epsilon_{2}}{\partial k}\right]{\text{e}}^{2\text{i}\gamma_{2}d}. \end{array}$$

Here, it can be seen that due to the presence of $\gamma_{2}=\sqrt{k_{0}^{2}\epsilon_{2}-k^{2}}$ , as long as medium 2 is nonlocal, the local contribution is formally nonlocal as well, being also contributed by the nonlocal term proportional to *∂ϵ*_2_/*∂k*. The latter does not exist if medium 2 is local, in which case the former is the only nonzero local contribution, thus justifying its name.

Equations similar to the above can also be obtained for the *s*-polarization. Redefining *A*_*ij*_ and *B*_*ij*_ of Eq. (26) as

(34)
$${\bar{A}}_{ij}=\gamma_{i}-\gamma_{j},\;\;\;\;\;{\bar{B}}_{ij}=\gamma_{i}+\gamma_{j},\;\;\;\;\;r_{s}^{ij}=\frac{{\bar{A}}_{ij}}{{\bar{B}}_{ij}},$$

one obtains

(35)
$$\frac{\partial r_{s}^{ij}}{\partial k}={\left(\frac{\partial r_{s}^{ij}}{\partial k}\right)}_{\text{loc}}+{\left(\frac{\partial r_{s}^{ij}}{\partial k}\right)}_{\text{nloc}}$$

with

(36)
$$\begin{array}{c} {\left(\frac{\partial r_{s}^{ij}}{\partial k}\right)}_{\text{loc}}=-\frac{{\bar{A}}_{ij}}{{\bar{B}}_{ij}^{2}}{\bar{B}}_{ij,loc}^{\prime }+\frac{1}{{\bar{B}}_{ij}}{\bar{A}}_{ij,loc}^{\prime },\\ {\left(\frac{\partial r_{s}^{ij}}{\partial k}\right)}_{\text{s,nloc}}=-\frac{{\bar{A}}_{ij}}{{\bar{B}}_{ij}^{2}}{\bar{B}}_{ij,nloc}^{\prime }+\frac{1}{{\bar{B}}_{ij}}{\bar{A}}_{ij,nloc}^{\prime }, \end{array}$$

where

(37)
$${\bar{A}}_{ij,loc}^{\prime }=k\left(\frac{1}{\gamma_{j}}-\frac{1}{\gamma_{i}}\right),\;\;{\bar{A}}_{ij,nloc}^{\prime }=\frac{\partial \epsilon_{i}}{\partial k}\frac{k_{0}^{2}}{2\gamma_{i}}-\frac{\partial \epsilon_{j}}{\partial k}\frac{k_{0}^{2}}{2\gamma_{j}},$$

(38)
$${\bar{B}}_{ij,loc}^{\prime }=-k\left(\frac{1}{\gamma_{j}}+\frac{1}{\gamma_{i}}\right),\;\;{\bar{B}}_{ij,nloc}^{\prime }=\frac{\partial \epsilon_{i}}{\partial k}\frac{k_{0}^{2}}{2\gamma_{i}}+\frac{\partial \epsilon_{j}}{\partial k}\frac{k_{0}^{2}}{2\gamma_{j}}.$$

This gives

(39)
$$\frac{\partial R_{s}}{\partial k}={\left(\frac{\partial R_{s}}{\partial k}\right)}_{\text{loc}}+{\left(\frac{\partial R_{s}}{\partial k}\right)}_{\text{nloc}}$$

with

(40)
$$\begin{array}{c} {\left(\frac{\partial R_{s}}{\partial k}\right)}_{\text{loc}}=-\frac{\bar{C}}{{\bar{D}}^{2}}{\bar{D}}_{\text{loc}}^{\prime }+\frac{1}{\bar{D}}{\bar{C}}_{\text{loc}}^{\prime },\\ {\left(\frac{\partial R_{s}^{ij}}{\partial k}\right)}_{\text{nloc}}=-\frac{\bar{C}}{{\bar{D}}^{2}}{\bar{D}}_{\text{nloc}}^{\prime }+\frac{1}{\bar{D}}{\bar{C}}_{\text{nloc}}^{\prime }, \end{array}$$

where

(41)
$$\bar{C}=r_{s}^{12}+r_{s}^{23}{\text{e}}^{2\text{i}\gamma_{2}d},\;\;\;\;\;\bar{D}=1+r_{s}^{12}r_{s}^{23}{\text{e}}^{2\text{i}\gamma_{2}d}$$

and

(42)
$$\begin{array}{ll} {\bar{C}}_{\text{loc}}^{\prime } & ={\left(r_{s}^{12}\right)}_{\text{loc}}^{\prime }+{\left(r_{s}^{23}\right)}_{\text{loc}}^{\prime }{\text{e}}^{2\text{i}\gamma_{2}d}-r_{s}^{23}{\text{e}}^{2\text{i}\gamma_{2}d}\frac{2\text{i}kd}{\gamma_{2}},\\ {\bar{C}}_{\text{nloc}}^{\prime } & ={\left(r_{p}^{12}\right)}_{\text{nloc}}^{\prime }+{\left(r_{s}^{23}\right)}_{\text{nloc}}^{\prime }{\text{e}}^{2\text{i}\gamma_{2}d}+r_{s}^{23}{\text{e}}^{2\text{i}\gamma_{2}d}\frac{\text{i}k_{0}^{2}d}{\gamma_{2}}\frac{\partial \epsilon_{2}}{\partial k}, \end{array}$$

(43)
$$\begin{array}{ll} {\bar{D}}_{\text{loc}}^{\prime } & =\left[{\left(r_{s}^{12}\right)}_{\text{loc}}^{\prime }r_{s}^{23}+r_{s}^{12}{\left(r_{s}^{23}\right)}_{\text{loc}}^{\prime }-r_{s}^{12}r_{s}^{23}\frac{2\text{i}k}{\gamma_{2}}\right]{\text{e}}^{2\text{i}\gamma_{2}d},\\ {\bar{D}}_{\text{nloc}}^{\prime } & =\left[{\left(r_{s}^{12}\right)}_{\text{nloc}}^{\prime }r_{s}^{23}+r_{s}^{12}{\left(r_{s}^{23}\right)}_{\text{nloc}}^{\prime }-r_{s}^{12}r_{s}^{23}\frac{\text{i}k_{0}^{2}}{\gamma_{2}}\frac{\partial \epsilon_{2}}{\partial k}\right]{\text{e}}^{2\text{i}\gamma_{2}d}. \end{array}$$

The set of equations above is used to numerically evaluate the GH shifts.

## Appendix B: Classification of EM modes

### B.1 Preserved in-plane reflection symmetry: TiN film free standing in air

Here, we analyze the EM modes of a finite-thickness TiN film free standing in air, in order to be able to identify those contributing to the GH shifts. In what follows, the “prime”-sign (′) and “double prime”-sign (″) abbreviate the real Re(…) part and imaginary Im(…) part, respectively.

We start with the interface EM modes between two infinitely extended media [48]. They are the Brewster mode and the surface mode. Both of them can be present at the two air/TiN interfaces separating media 1 and 3 (air above and below the TiN film) from medium 2 (the film itself), and both are described by the condition

(44)
$$k=\frac{\omega }{c}\sqrt{\frac{\epsilon_{\text{TiN}}}{\epsilon_{\text{TiN}}+1}}.$$

This can be obtained from either $r_{p}^{12}=-r_{p}^{23}=0$ with $\epsilon_{\text{TiN}}^{\prime }\geq 0$ (*ω* ≥ *ω*_*p*_) or $r_{p}^{12}=-r_{p}^{23}=\infty$ with $\epsilon_{\text{TiN}}^{\prime }<0$ (*ω* < *ω*_*p*_) in Eq. (20), to give for media 1 and 3 (air) the Brewster mode in the propagating wave region *k* ≤ *ω*/*c* and the surface mode in the evanescent wave region *k* > *ω*/*c*, respectively. Both of them lead to *R*_*p*_ = 0 according to Eq. (20) and thus to the stronger GH effect as per Eq. (19). To obtain the corresponding dispersion relations, Eq. (44) should be solved with dissipation included for either the complex valued *k* at a given real frequency *ω* or the complex valued *ω* at a given real valued in-plane momentum *k*. Depending on the physical situation, either approach has to be used [48].

Neglecting the dissipation in Eq. (44) leads to the idealized solution with real valued *k* and *ω*, which for nonlocal *ϵ*_TiN_(*ω*, *k*) of Eqs. (3)–(5) with Γ_*D*_ = 0 takes the following form

(45)
$$\begin{array}{ll} \omega^{2} & =\frac{1}{2}\left[k^{2}c^{2}\frac{\epsilon_{b}+1}{\epsilon_{b}}+\omega_{p}^{2}\left(k\right)\right]\\ & \;\pm \sqrt{\frac{1}{4}{\left[k^{2}c^{2}\frac{\epsilon_{b}+1}{\epsilon_{b}}+\omega_{p}^{2}\left(k\right)\right]}^{2}-k^{2}c^{2}\omega_{p}^{2}\left(k\right)}. \end{array}$$

Here, the +(−) sign solution describes the Brewster (surface) mode in the propagating (evanescent) wave region. With $\omega_{p}^{2}\left(k\right)$ of Eq. (4), in the limit *d* → ∞, the well-known local dispersion relations can be recovered [48].

In the finite-thickness films, in addition to the interface modes, there are also standing waves to represent the eigenmodes of the film itself. The standing wave modes are responsible for enhanced transmission and so reduced reflection of the film. For our nonlocal free standing TD plasmonic film of thickness *d*, the standard standing wave condition is *γ*_2_ = *πn*/*d* with n∈N , the same as that studied for polaritonic waves in thin dielectric films previously [49], yielding

(46)
$$k=\sqrt{\frac{\omega^{2}}{c^{2}}\epsilon_{\text{TiN}}-{\left(\frac{\pi n}{d}\right)}^{2}},$$

to give

(47)
$$\omega =\sqrt{\frac{k^{2}c^{2}}{\epsilon_{b}}+\omega_{p}^{2}\left(k\right)+\frac{c^{2}}{\epsilon_{b}}{\left(\frac{\pi n}{d}\right)}^{2}}$$

for *ϵ*_TiN_(*ω*, *k*) of Eqs. (3)–(5) with dissipation neglected as before. Again, in the limit *d* → ∞, one obtains the local version of the standing wave solutions in the TD plasmonic film. These solutions are similar but not exactly the same as those reported previously for the local standing polaritonic waves [49] due to the different in-plane EM responses of the metallic and dielectric thin films.

It should be noted that Eq. (47) can be generalized to include dissipative effects, too, if one starts from the constraint |*R*_*p*_|^2^ = 0 as given by Eq. (20) with $r_{p}^{12}=-r_{p}^{23}$ canceled out to exclude the interface modes already discussed. Then,

(48)
$$1+{\text{e}}^{2\text{i}\left(\gamma_{2}-\gamma_{2}^{*}\right)d}=2\text{Re}\left({\text{e}}^{-2\text{i}\gamma_{2}^{*}d}\right),$$

which can be brought to the trigonometrical form

$$\sin\left[\left(\gamma_{2}^{\prime }+\gamma_{2}^{\prime \prime }\right)d\right]\sin\left[\left(\gamma_{2}^{\prime }-\gamma_{2}^{\prime \prime }\right)d\right]=0$$

yielding

(49)
$$\left(\gamma_{2}^{\prime }\pm \gamma_{2}^{\prime \prime }\right)d=\pi n,\;\;\;n=0,\pm 1,\pm 2,\pm 3,\ldots ,$$

where $\gamma_{2}^{\prime }$ and $\gamma_{2}^{\prime \prime }$ , the real and imaginary parts of *γ*_2_, can be obtained from its complex exponential form

$$\begin{array}{ll} \gamma_{2} & =k_{0}\sqrt{\epsilon_{\text{TiN}}-{\left(k/k_{0}\right)}^{2}}=\gamma_{2}^{\prime }+\text{i}\gamma_{2}^{\prime \prime }\\ & =|\gamma_{2}|\;{\text{e}}^{\text{i}\left(Arg\left(\gamma_{2}\right)+2\pi m\right)/2},\;m=0,1,\\ \left|\gamma_{2}\right| & =k_{0}{\left\{{\left[\epsilon_{\text{TiN}}^{\prime }-{\left(k/k_{0}\right)}^{2}\right]}^{2}+\epsilon_{\text{TiN}}^{\prime \prime \;2}\right\}}^{\;1/4},\\ Arg\left(\gamma_{2}\right) & =\arctan\;\left[\frac{\epsilon_{\text{TiN}}^{\prime \prime }}{\epsilon_{\text{TiN}}^{\prime }-{\left(k/k_{0}\right)}^{2}}\right]. \end{array}$$

Plugging them in Eq. (49) leads after straightforward simplifications to the transcendental equation as follows

(50)
$$\begin{array}{cc} & \sin\left\{\frac{1}{2}\arctan\left[\frac{\epsilon_{\text{TiN}}^{\prime \prime }}{\epsilon_{\text{TiN}}^{\prime }-{\left(k/k_{0}\right)}^{2}}\right]\pm \frac{\pi }{4}\right\}\\ & \;=\frac{\pi n}{\sqrt{2}\;k_{0}d\;{\left\{{\left[\epsilon_{\text{TiN}}^{\prime }-{\left(k/k_{0}\right)}^{2}\right]}^{2}+\epsilon_{\text{TiN}}^{\prime \prime \;2}\right\}}^{\;1/4}},\\ & \;n=0,\pm 1,\pm 2,\pm 3,\ldots \end{array}$$

The transcendental equation (50) sets up the EM modes of the TD film that are responsible for its zero reflection and thus for either enhanced transmission or enhanced absorption of external EM radiation incident on the film. It can be solved for *ω* analytically. In the negligible dissipation case, one has $\epsilon_{\text{TiN}}^{\prime \prime }\ll |\epsilon_{\text{TiN}}^{\prime }-{\left(k/k_{0}\right)}^{2}|$ to obtain

$$k_{0}\sqrt{\left|\epsilon_{\text{TiN}}^{\prime }-{\left(k/k_{0}\right)}^{2}\right|}=\frac{\pi n}{d},\;\;\;n\in N,$$

which after plugging $\epsilon_{\text{TiN}}^{\prime }\left(\omega ,k\right)$ in it leads to the generalized form of Eq. (47) as follows

(51)
$$\omega =\sqrt{\frac{k^{2}c^{2}}{\epsilon_{b}}+\omega_{p}^{2}\left(k\right)+\text{sign}\;\left[\omega^{2}-\omega_{p}^{2}\left(k\right)-{\left(kc/\;\sqrt{\epsilon_{b}}\right)}^{2}\right]\frac{c^{2}}{\epsilon_{b}}{\left(\frac{\pi n}{d}\right)}^{2}},\;\;\;n\in N.$$

This includes both propagating waves of Eq. (47) and evanescent waves as well, in medium 2 (TiN film), which come out as solution branches with $\omega^{2}>\omega_{p}^{2}+{\left(kc/\sqrt{\epsilon_{b}}\right)}^{2}$ and $\omega^{2}<\omega_{p}^{2}+{\left(kc/\sqrt{\epsilon_{b}}\right)}^{2}$ , respectively. In the strong dissipation case, where $\epsilon_{\text{TiN}}^{\prime \prime }\gg |\epsilon_{\text{TiN}}^{\prime }-{\left(k/k_{0}\right)}^{2}|$ , the properties of the arctangent allow one to rewrite Eq. (50) in the form

$$\sin\left\{\frac{\pi }{4}\;\text{sign}\left[\epsilon_{\text{TiN}}^{\prime }-{\left(k/k_{0}\right)}^{2}\right]\pm \frac{\pi }{4}\right\}=\frac{\pi n}{k_{0}d\sqrt{2\epsilon_{\text{TiN}}^{\prime \prime }}},$$

with the only legitimate solution (the solution that stays asymptotically correct as *d* → ∞) given by $\epsilon_{\text{TiN}}^{\prime }-{\left(k/k_{0}\right)}^{2}=0$ and *n* = 0. With our nonlocal $\epsilon_{\text{TiN}}^{\prime }\left(\omega ,k\right)$ of Eqs. (3)–(5), this yields the dispersion equation for the fundamental plasma mode of the finite-thickness TD film

$$\omega =\sqrt{\frac{k^{2}c^{2}}{\epsilon_{b}}+\omega_{p}^{2}\left(k\right)},\;\;\;n=0,$$

which can be combined with Eq. (51) to give the final standing wave solution as follows

(52)
$$\omega =\sqrt{\frac{k^{2}c^{2}}{\epsilon_{b}}+\omega_{p}^{2}\left(k\right)+\text{sign}\;\left[\omega^{2}-\omega_{p}^{2}\left(k\right)-{\left(kc/\;\sqrt{\epsilon_{b}}\right)}^{2}\right]\frac{c^{2}}{\epsilon_{b}}{\left(\frac{\pi n}{d}\right)}^{2}},\;\;\;n=0,1,2,3,\ldots$$

However, one has to remember that here, contrary to the *n* ≠ 0 low-dissipative modes of Eq. (51), the fundamental mode with *n* = 0 is associated with strong dissipation of EM radiation absorbed by the TD film to generate the in-plane plasma waves in the system.

Figure 8 shows and describes in the caption the features of the propagating and evanescent wave dispersion relations given by Eq. (52) with *n* = 0, 1, 2, and 3, presented in the dimensionless (*ω*, *k*)-space for a few free standing TiN films of decreasing thickness. The Brewster and surface modes of Eq. (45) are also shown. Note that due to the in-plane reflection symmetry of the free standing TD film system, all modes shown lead to zero *p*-wave reflection coefficient in Eq. (20) as it follows from the discussion above. The detailed analysis and general properties of the evanescent wave solutions in TD plasmonic films can be found in Refs. [26], [28]. The GH shift calculations require the knowledge of the propagating wave solutions, which we are, therefore, focusing on below.

Figures 9 and 10 show the calculated inverse reflectivities and discuss in the captions some of the propagating wave dispersion relations in the (*ω*, *θ*_*i*_)-space, described by local (Drude) and nonlocal KR in-plane EM response functions as given by Eqs. (3) and (4) for infinitely large and finite *d*, respectively. It can be seen that the dispersion relations of Eqs. (45) and (47) for the Brewster modes and standing wave modes with losses neglected are in full agreement with direct numerical calculations of Eq. (20) including losses. It can also be seen that at small angles of incidence, i.e., for small *k*, the nonlocality of the in-plane EM response plays an important role. More features in the (*ω*, *θ*_*i*_)-space can be seen in Figures 11–14. Figures 11 and 12 show the phases (normalized by *π*) of the reflection coefficients whose inverse squares are shown in Figures 9 and 10, respectively. Figures 13 and 14 present the respective GH shifts, where it can be seen that due to the EM response nonlocality, the large GH shifts can be obtained for frequencies below the bulk plasma frequency $\omega_{p}^{3D}$ , for example, by using He-Ne laser light.

### B.2 Broken in-plane reflection symmetry: TiN film on MgO substrate

In this case, there are two different (inequivalent) interfaces with their respective interface modes. Our TD plasmonic TiN film (medium 2) is now sandwiched between air (medium 1) and a MgO substrate with *ϵ*_MgO_ = 3.0 (medium 3). The top-bottom interface mode degeneracy is lifted as compared to the free standing plasmonic film case. However, we show in what follows that the propagating standing waves of the film can still provide zero reflection given that proper Brewster mode constraints are fulfilled at both of the inequivalent interfaces, which is possible at mode intersection points in the (*ω*, *k*)-space. They are the (topological) phase singularity points to replace the lines of the free standing film case and thus to greatly enhance the GH effect.

For the air/TiN interface Brewster and surface modes one still has Eq. (45), whereas for the MgO/TiN interface

(53)
$$\begin{array}{ll} \omega^{2} & =\frac{1}{2}\left(k^{2}c^{2}\frac{\epsilon_{b}+\epsilon_{3}}{\epsilon_{b}\epsilon_{3}}+\omega_{p}^{2}\left(k\right)\right)\\ & \;\pm \sqrt{\frac{1}{4}{\left(k^{2}c^{2}\frac{\epsilon_{b}+\epsilon_{3}}{\epsilon_{b}\epsilon_{3}}+\omega_{p}^{2}\left(k\right)\right)}^{2}-\frac{k^{2}c^{2}\omega_{p}^{2}\left(k\right)}{\epsilon_{3}}}, \end{array}$$

where +(−) correspond to the Brewster (surface) mode in the propagating (evanescent) wave region of medium 3 and *ϵ*_3_ = *ϵ*_MgO_. The propagating modes of the TiN film itself are still given by the standing wave solutions of Eq. (47). However, contrary to thick films where $\omega_{p}\left(k\right)∼\omega_{p}^{3D}$ is the same for all modes, for the TD plasmonic film system $\omega_{p}\left(k\right)∼\omega_{p}^{3D}\sqrt{kd\epsilon_{b}/\left(\epsilon_{1}+\epsilon_{3}\right)}$ as per Eq. (4), which is different from *ω*_*p*_(*k*) of the free standing TD film case.

All modes of relevance to the GH effect in our system can be summarized as follows:

(1.) The air/TiN interface Brewster mode described by the + sign branch of Eq. (45), to yield $r_{\text{p}}^{12}=0$ ;
(2.) The MgO/TiN interface Brewster mode described by the + sign branch of Eq. (53), to yield $r_{\text{p}}^{23}=0$ ;
(3.) The propagating modes of the TiN film given by Eq. (52), originating from Eq. (48) with $\gamma_{2}^{\prime \prime }=0$ , to yield $1-{\text{e}}^{2\text{i}\gamma_{2}d}=0$ for *γ*_2_ = *πn*/*d* with *n* = 0, 1, 2, …;
(4.) For TD films, of significance can also be the Brewster mode of a hypothetical interface between medium 1 and medium 3, in which case by analogy with Eq. (44) one has $k=\left(\omega /c\right)\sqrt{\epsilon_{3}/\left(\epsilon_{3}+1\right)}$ , or $\omega =kc\sqrt{\left(\epsilon_{3}+1\right)/\epsilon_{3}}$ , to yield $r_{\text{p}}^{13}=0$ ;
(5.) From Eq. (20), it can be seen that $\epsilon_{2}^{\prime }\left(k\right)=1$ (air/TiN interface Christiansen point), or $\omega =\omega_{p}\left(k\right)\sqrt{\epsilon_{b}/\left(\epsilon_{b}-1\right)}$ as per Eq. (4), leads to $r_{\text{p}}^{12}=0$ ;
(6.) Similarly, $\epsilon_{2}^{\prime }\left(k\right)=\epsilon_{3}$ (MgO/TiN interface Christiansen point), or $\omega =\omega_{p}\left(k\right)\sqrt{\epsilon_{b}/\left(\epsilon_{b}-\epsilon_{3}\right)}$ , leads to $r_{\text{p}}^{23}=0$ .

There is a simple method to find the zeroes of the reflection coefficient in Eq. (20). As they come from the numerator

(54)
$$N\left(\omega ,k\right)=r_{p}^{12}+r_{p}^{23}{\text{e}}^{2\text{i}\gamma_{2}d},$$

to find them we start with the Brewster mode of a hypothetical interface between medium 1 and 3 (air/MgO) mentioned in (4.) above. This is the case where *γ*_1_ *ϵ*_3_ = *γ*_3_ *ϵ*_1_. Using this inside $r_{p}^{13}=0$ leads to $r_{p}^{12}=-r_{p}^{23}$ , now for TD plasmonic film systems with broken in-plane reflection symmetry. This is possible because $r_{p}^{12}$ and $r_{p}^{23}$ are functions of *ω* and *k*, and their equality implies nothing but their intersection point in the (*ω*, *k*)-space as opposed to their exact coincidence (identity) in the degenerate case of the preserved in-plane reflection symmetry of free standing TD films. Hence, one has

(55)
$$N\left(\omega ,k\right)=r_{p}^{12}+r_{p}^{23}{\text{e}}^{2\text{i}\gamma_{2}d}=r_{p}^{23}\left(-1+{\text{e}}^{2\text{i}\gamma_{2}d}\right).$$

This expression is now zero in the following cases:

(A) If we assume that $r_{p}^{13}=0$ and *γ*_1_ *ϵ*_3_ = *γ*_3_ *ϵ*_1_ are fulfilled, then *N*(*ω*, *k*) = 0 if $r_{p}^{23}=0$ . That means the reflection coefficient *R*_*p*_ can only be zero at the crossing points of the Brewster mode of the air/MgO interface from (4.) and the Brewster mode of the MgO/TiN interface from (2.).
(B) If we assume that $r_{p}^{13}=0$ and *γ*_1_ *ϵ*_3_ = *γ*_3_ *ϵ*_1_ are fulfilled, then *N*(*ω*, *k*) = 0 if $\left(-1+{\text{e}}^{2\text{i}\gamma_{2}d}\right)=0$ . That means the reflection coefficient *R*_*p*_ can only be zero at the crossing points of the Brewster mode of the air/MgO interface from (4.) and the standing wave modes from (3.).
(C) From the condition (A) that when $r_{p}^{13}=0$ and *γ*_1_ *ϵ*_3_ = *γ*_3_ *ϵ*_1_ are fulfilled and $r_{p}^{23}=0$ , then *R*_*p*_ = 0, it follows also that when $r_{p}^{13}=0$ and $r_{p}^{12}=0$ are fulfilled there is zero of *R*_*p*_, because $r_{p}^{23}=-r_{p}^{12}$ . This means that when the Brewster mode of the air/MgO interface from (4.) and the Brewster mode of the air/TiN interface from (1.) cross then there is a zero reflection point, too.

Additionally, one has:

(D) Since $r_{p}^{12}=0$ is also fulfilled for the Christiansen point in (5.), there is another possible zero at the crossing point of the Christiansen mode from (5.) and the Brewster mode of the MgO/TiN interface from (2.).
(E) Since $r_{p}^{23}=0$ is also fulfilled for the Christiansen point in (6.), there is another possible zero at the crossing point of the Christiansen mode from (6.) and the Brewster mode of the MgO/TiN interface from (2.).
(F) The above solutions come from either intersection of the Brewster and Christiansen modes or the intersection of the Brewster mode of the (hypothetical) medium 1/medium 3 interface and the standing waves of medium 2. On closer inspection of Eq. (54), however, one finds another class of solutions, which is associated not with the standing waves fulfilling $\left(-1+{\text{e}}^{2\text{i}\gamma_{2}d}\right)=0$ but with those fulfilling $\left(1+{\text{e}}^{2\text{i}\gamma_{2}d}\right)=0$ . This comes from the constraint $r_{p}^{12}=r_{p}^{23}$ , which can be the case when $\gamma_{2}^{2}\epsilon_{1}\epsilon_{3}=\epsilon_{2}^{2}\gamma_{3}\gamma_{1}$ . It leads to the analogue of Eq. (47) with *n* = 0.5, 1.5, 2.5, …

Figure 15 shows and comments in the caption on how cases (A), (B), and (C) can be understood in terms of the mode intersection points in the (*ω*, *k*)-space. Figures 16 and 17 show and comment on the above listed singularity locations in the inverse reflectivity and reflection coefficient phase in the (*ω*, *θ*_*i*_)-space.

Note that cases (B) and (F) above are referred to as cases 1 and 2 in the main text. In these two cases, the phase singularities are defined by the intersection of the standing wave mode dispersion curves with the two different Brewster mode dispersion curves. Note also that in our configuration, cases (C) and (E) coincide with the energetically lowest phase singularity of case 1 in the main text, or (B) here, which is red-shifted for thinner films due to the nonlocal in-plane EM response effect. Moreover, cases (A) and (D) herein define another phase singularity not covered by cases (B) and (F), or cases 1 and 2 in the main text.

Finally, we would like to stress that the Brewster modes discussed here to provide the most important singularity points are those given by the reflection coefficient zeros (also known as improper modes [50]), to which, therefore, it is impossible to assign a group velocity. In contrast, the surface modes are those given by the poles of the reflection coefficient [26]. They are the proper eigenmodes confined to the interface [28], to which one can assign both phase and group velocity. The differences between the two can be clearly seen in Figures 8 and 15 for the TD films with and with no in-plane reflection symmetry, respectively.
